# Reconstructing growth and dynamic trajectories from single-cell transcriptomics data

**DOI:** 10.1038/s42256-023-00763-w

**Published:** 2023-11-30

**Authors:** Yutong Sha, Yuchi Qiu, Peijie Zhou, Qing Nie

**Affiliations:** 1 Department of Mathematics, University of California, Irvine, Irvine, CA USA; 2 Department of Mathematics, Michigan State University, East Lansing, MI USA; 3 Department of Developmental and Cell Biology, University of California, Irvine, Irvine, CA USA; 4 The NSF-Simons Center for Multiscale Cell Fate Research, University of California, Irvine, Irvine, CA USA

**Keywords:** Machine learning, Data integration

## Abstract

Time-series single-cell RNA sequencing (scRNA-seq) datasets provide unprecedented opportunities to learn dynamic processes of cellular systems. Due to the destructive nature of sequencing, it remains challenging to link the scRNA-seq snapshots sampled at different time points. Here we present TIGON, a dynamic, unbalanced optimal transport algorithm that reconstructs dynamic trajectories and population growth simultaneously as well as the underlying gene regulatory network from multiple snapshots. To tackle the high-dimensional optimal transport problem, we introduce a deep learning method using a dimensionless formulation based on the Wasserstein–Fisher–Rao (WFR) distance. TIGON is evaluated on simulated data and compared with existing methods for its robustness and accuracy in predicting cell state transition and cell population growth. Using three scRNA-seq datasets, we show the importance of growth in the temporal inference, TIGON’s capability in reconstructing gene expression at unmeasured time points and its applications to temporal gene regulatory networks and cell–cell communication inference.

## Abstract (web summary)

Single-cell transcriptomics has provided a powerful approach to investigate cellular properties at unprecedented resolution. Sha et al. have developed an optimal transport-based algorithm called TIGON that can connect transcriptomic snapshots from different time points to obtain collective dynamical information, including cell population growth and the underlying gene regulatory network.

## Main

Single-cell RNA sequencing (scRNA-seq) methods offer a systematic and scalable approach to observing dynamics by sampling cells at different times^1^. However, cells are killed during sequencing and time-series scRNA-seq only provides unpaired snapshots. As a result, the cell lineage relationship or cell trajectory between different sequenced times is missing and gene expression dynamics of individual cells are not traceable. Lineage tracing combined with scRNA-seq can reveal clonal relationships; however, it lacks single-cell resolution and is limited to in vitro in most cases^2–6^.

Pseudotime orders cells along differentiation trajectories, based on the assumption that developmentally related cells share similarities in gene expression^7–11^. RNA velocity utilizes the spliced-to-unspliced mRNA ratio to infer the cell transition direction^12^. Population balance analysis employs spectral graph theory to represent gene expression dynamics when the cellular system is under steady state^13^. The dynamical systems approach provides a natural way for reconstructing trajectory and velocity^14,15^. CoSpar infers a transition map by using additional experimental temporal clonal information^16^. Dynamo reconstructs continuous velocity fields of cell transitions by modelling unspliced and spliced counts from time-resolved metabolic labelling data^17^. PRESCIENT learns differentiation landscapes by modelling cell differentiation as diffusion^18^. MuTrans utilizes multiscale reduction to quantify attractors and their transition probabilities in snapshot data, as well as constructing a low-dimensional dynamical manifold^19^. However, these methods usually assume stationarity or equilibrium^19,20^, and cannot capture temporally evolving dynamics, such as development. The Fokker–Planck equation can be used for cell population dynamics^21^, but it is challenging to infer the parameters and solve the equations efficiently.

Optimal transport (OT), a classic mathematical theory on transporting masses between two distributions^22^, has been recently used for time-series scRNA-seq measurements. Waddington-OT considers cells drawn from a probability distribution in gene expression space and uses OT to infer transport plans between two consecutive time points^23^. Another formulation of OT, known as dynamic OT, where the addition of time gives an alternative interpretation with links to fluid dynamics, surprisingly leads to a convex optimization problem^24^. TrajectoryNet connects dynamic OT and continuous normalizing flows to infer continuous paths of cellular dynamics^25^. MIOFlow uses a geodesic autoencoder (AE) and a multiscale manifold distance to learn stochastic dynamics of snapshots by implementing OT flows on a data manifold^26^.

**Fig. 1:**
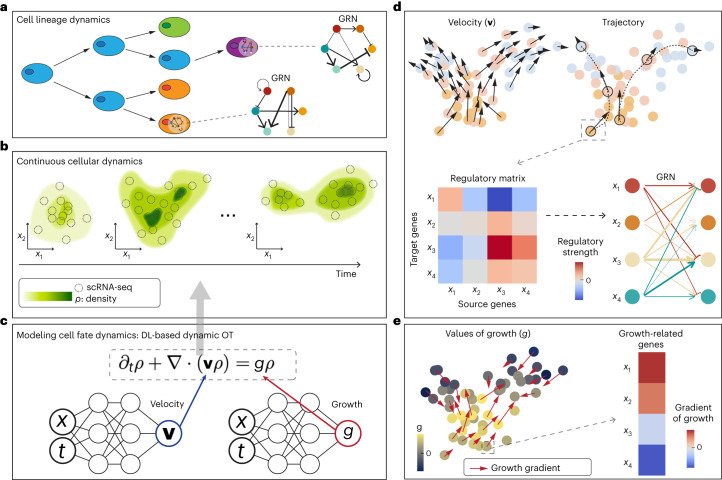
Illustrative diagram of TIGON. **a**, Illustrative graph of cell lineage dynamics which involves cell growth, transition and GRNs. **b**, The continuous cellular dynamics are described by a time-dependent density *ρ*(*x*,*t*). The input of time-series scRNA-seq snapshots generates density *ρ* at discrete time points. **c**, The density *ρ* is governed by a partial differential equation involving velocity **v** and growth *g* that are modelled by two neural networks. DL, deep learning. **d**,**e**, Outputs and downstream analysis of TIGON. **d**, Top left, velocity, where each dot represents a cell coloured by collection time and length of arrow denotes the magnitude of the velocity. Top right, trajectory of each cell. Bottom left, gene regulatory matrix of a selected cell or cell type. Bottom right, GRN, where the pointed arrows (blunt arrows) denote positive (negative) regulation from the source gene to the target gene and the width denotes regulatory strength. **e**, Left, inferred values of growth *g* are represented by colour. The red arrow denotes the gradient of *g* with its length corresponding to the magnitude. Right, the gradient of *g* determines the contributions of genes to the growth changes. Growth-related genes are selected based on those with the largest gradient.

In such a model, the concept of velocity is introduced to describe the instantaneous change in gene expression over time for each cell. Because cell populations may change in time due to cell division and cell death (Fig. 1a), a growth term that captures such net change may be needed in the model. It is increasingly clear that without incorporating growth, the inferred dynamics for cell trajectory are often incomplete and less accurate^13,27,28^. Pioneering works, such as Waddington-OT and PRESCIENT, utilize growth hallmark gene expression to approximate growth^18,23^. However, the knowledge bases such as the Kyoto Encyclopedia of Genes and Genomes (KEGG)^29^ and Gene Ontology (GO)^30^ may provide distinct gene lists and the inferred growth exhibits considerable dependence on database-selection, as demonstrated in the present study. TrajectoryNet is the first method to consider growth/death by incorporating it as a separate discrete static unbalanced OT model in the continuous setting^25^. Despite these advances, models and computational tools that can incorporate both gene expression velocity of each cell and cell population growth simultaneously are currently lacking.

Here we propose TIGON (Trajectory Inference with Growth via Optimal transport and Neural network) that infers cell velocity, growth and cellular dynamics by connecting unpaired time-series single-cell transcriptomics data. TIGON is a dynamic, unbalanced OT model. The method is based on Wasserstein–Fisher–Rao (WFR) distance, generalizing OT to measures of different masses^31–33^. The approach consists of three unique features: (1) a dynamic unbalanced OT model that can simultaneously capture the velocity of gene expression for each cell and the cell population over time, (2) a mesh-free, dimensionless formulation based on WFR distance that is readily solvable by neural ordinary differential equations (ODEs) and (3) inference of temporal, causal gene regulatory networks (GRNs) and growth-related genes.

Through a simulated gene regulatory model, we show the utility of TIGON in modelling cell velocity and growth in a unified framework by comparing it to the balanced dynamic OT model. We further test and compare TIGON on three time-series systems including a lineage tracing dataset with bifurcation, an epithelial-to-mesenchymal transition (EMT) dataset and an induced pluripotent stem cell (iPSC) differentiation dataset with bifurcation. TIGON accurately recovers the velocity, trajectory and growth of cells, in addition to inferring temporal GRNs and cell–cell communication.

## Results

### Overview of TIGON

In the model, a group of cells is described by a time-dependent density *ρ*(*x,t*), where *ρ*(*x,t*) is the distribution of cell number over gene expression state *x* at a time *t*. The gene expression state $x\in {{\mathbb{R}}}^{d}$ is in the *d*-dimensional gene expression space ${{\mathbb{R}}}^{d}$. Time-series scRNA-seq data is used to generate density functions at the given discrete time points: *ρ*_*i*_ = *ρ*(*x,t*_*i*_), *i* = 1, 2,⋯,*T* using a Gaussian mixture model (Fig. 1b and Methods). The deep learning-based method in TIGON reconstructs *ρ*(*x,t*), by interpolating the input time-series densities *ρ*_*i*_ using a hyperbolic partial differential equation (Fig. 1c):^13,21,28,31,32^

1 $${\partial }_{t}\rho \left(x,t\right)+\nabla \cdot \left(\mathbf{v}\left(x,t\right)\rho \left(x,t\right)\right)=g\left(x,t\right)\rho \left(x,t\right).$$

The convection term ∇⋅(**v**(*x,t*)*ρ*(*x,t*)) describes the transport of cell density, and the velocity $\mathbf{v}\left(x,t\right)\in {{\mathbb{R}}}^{d}$ describes the instantaneous change of gene expression for cells in gene expression state *x* at time *t* (Fig. 1d). The growth term, *g*(*x,t*), describes the instantaneous population change (Fig. 1e). The velocity and growth together determine the cell density dynamics. Equation (1) is solved using unbalanced OT by optimizing the WFR cost^31,32^:

2 $${W}_{0,T}=T\mathop{\int}\limits_{0}^{T}\mathop{\int }\limits_{{{\mathbb{R}}}^{d}}\left({\left|\mathbf{v}\left(x,t\right)\right|}^{2}+\alpha {\left|g\left(x,t\right)\right|}^{2}\right)\rho \left(x,t\right){{\mathrm {d}}x\; {\mathrm {d}}t}.$$

The WFR distance was previously used for a fluid system utilizing quadratic Wasserstein and Fisher–Rao metrics to describe kinetic energy and energy of growth, respectively^31^. Solving equation (1) and minimizing objective function in equation (2) require computing high-dimensional integrals in gene expression space. To deal with the high dimensionality, we obtain a dimensionless formulation for the WFR-based dynamic unbalanced OT problem in equation (2) (Lemma and Theorem in Methods). Briefly, two neural networks are used to approximate velocity **v**(*x,t*) ≈ *NN*_1_(*x,t*) and growth *g*(*x,t*) ≈ *NN*_2_(*x,t*) (Fig. 1c). The formulation results in a system of ODEs, which is then solved and optimized by neural ODEs^34–36^ (Methods).

For cell trajectories, TIGON tracks the dynamics from progenitor to descendant state by integrating along the velocity field (Fig. 1d). The gene analysis describes how the state variable *x* (genes) interact and contribute to velocity and growth. This can be conducted at single-cell resolution or cell-type level by averaging the quantities over a group of cells to reduce randomness and enhance inference robustness. The GRN is constructed in a directed, signed and weighted graph with self-regulation from the regulatory matrix using the Jacobian of velocity $J={\left\{\frac{\partial \mathbf{v}_{i}}{\partial {x}_{j}}\right\}}_{i,\,j=1}^{d}$, where $\frac{\partial \mathbf{v}_{i}}{\partial {x}_{j}}$ describes the regulatory strength from source *j*-th gene to target *i*-th gene (Fig. 1d). In GRN, directions of edges illustrate the regulatory relation between source and target genes, and signs associated with these edges represent positive or negative regulation—activation or inhibition, respectively—that occurs between genes. The contribution of each gene to growth is assessed from the gradient of growth $\nabla g={\left\{\frac{\partial g}{\partial {x}_{j}}\right\}}_{j=1}^{d}$. The gradient of growth describes growth potential in the gene expression space, with the top ones defined as growth-related genes (Fig. 1e).

Each cell in the data usually contains thousands of genes. To efficiently use the TIGON method, we first perform dimension reduction, including uniform manifold approximation and projection (UMAP), principal component analysis (PCA) and an AE (Methods), to project the original data onto a low-dimensional space. The methods PCA and AE are reversible and differentiable, allowing for the direct approximation of the gradient of growth and computation of the regulatory matrix (Supplementary Note 1). TIGON requires the cell population at the measured time points as the input. When no prior information is given about cell population, we assume the cell population is represented by the number of cells collected at each time (Methods).

### Benchmark on a three-gene model

**Fig. 2:**
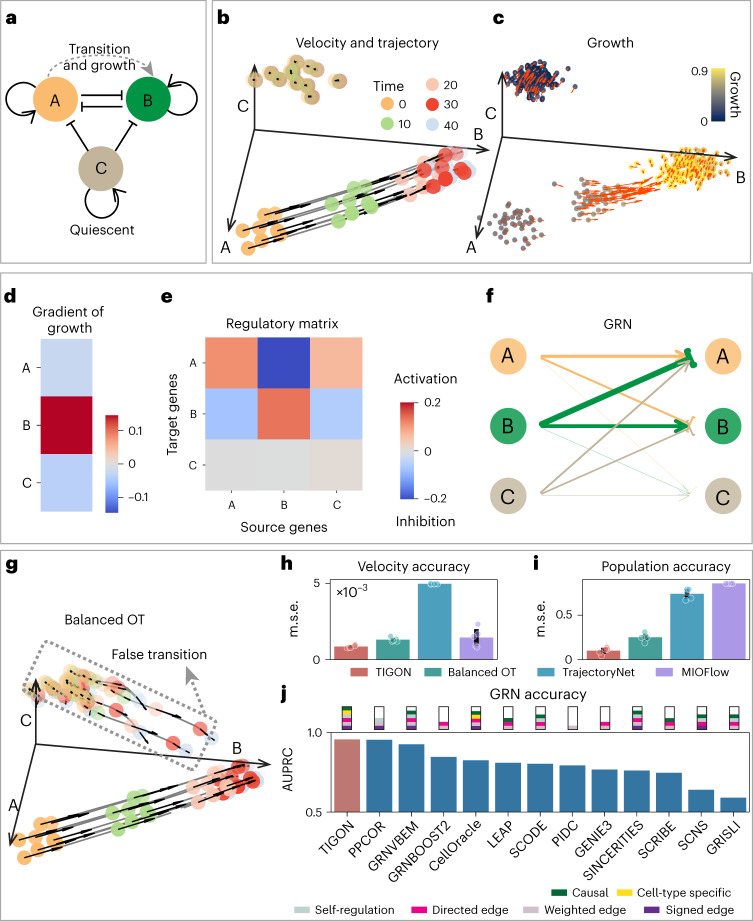
TIGON’s performance on three-gene simulated data. **a**, Illustrative diagram of the GRN of the three-gene model. **b**,**c**, Cellular dynamics for cells sampled at time = 0. **b**, Velocity and trajectory of cells. For each cell, its velocity is represented by black arrows, and its dynamic trajectory is represented by the grey curve. Here 20 randomly sampled cells from initial density at time = 0 are shown. **c**, Values of growth and gradient of growth. For each cell, the colour denotes its values of growth, and the red arrow shows its gradient of growth. At each time point, 100 sampled cells are shown. **d**–**f**, Gene analysis for transition cells at time = 5. **d**, Gradient of growth. **e**, Regulatory matrix. **f**, GRN displayed in a form of weighted directed graph. Pointed arrows (blunt arrows) denote the activation (inhibition) from the source gene at the starting point to the target gene at the end point. Width of lines denotes the regulatory strength. **g**, Velocity and trajectory inference from balanced OT by moving the growth term in TIGON. Identical 20 cells at time = 0 are selected as in **b**. **h**,**i**, Comparisons between TIGON and OT-based trajectory inference methods measured by accuracy in velocity predictions (**h**), and accuracy in predicting ratio of cell population (**i**) between transition cells and quiescent cells. The accuracy is measured by the m.s.e. The error bars show one standard deviation above and below the mean for each method from *n* = 5 independent repeats. Scatter plots show the accuracy from each repeat. **j**, Comparison of GRN inference methods. GRNs are calculated for transition cells at time = 0, 10, 20, …, 40. Barplots show the average GRN edge classification accuracy over these time points quantified by the area under precision-recall curve (AUPRC). Functionalities of each method are summarized in a rectangular box on the top of the bar.

We first tested various functionalities of TIGON and performed comparisons with several other existing methods for trajectory inference or GRN inference. We used an in-silico stochastic model based on a three-gene GRN, which consists of three cell states (Fig. 2a and Methods). The simulation generates two groups of cells with distinct cell dynamics (Supplementary Fig. 1a). One group of cells with highly expressed gene C remains static over time, illustrating a quiescent state that is vital for maintaining tissue balance. Another group of cells, initially in state A, undergoes a transition to state B. During transition, gene B upregulates cell division to enhance population growth.

Using five snapshots of simulated data (Supplementary Fig. 1a), TIGON identifies two groups of cells (Fig. 2b) and growth (Fig. 2c) that are consistent with the ground truth (Supplementary Fig. 1a,b). The velocity and gradient of growth show consistent directions and potentials of cells under transition from state A to state B, indicating the cooperative effects between velocity and growth in governing cellular dynamics (Fig. 2b,c). The gene analysis identifies gene B as the only gene that upregulates growth (Fig. 2d), and reconstructs the cell-type specific GRNs for cells under transition from A state to B state (Fig. 2e,f and Supplementary Fig. 2a,b). For cells undergoing transition, gene A and gene B are found to strongly inhibit each other, while gene C shows negligible regulatory strengths and unchanged expression near zero. TIGON correctly identifies the toggle-switch interactions between gene A and B.

Next, we compared TIGON with three OT-based trajectory inference methods (Fig. 2g–i). As the transition cells divide, the ratio of transition cells over quiescent cells increases (Supplementary Fig. 1c). Because of the incorporation of growth and velocity, TIGON accurately captures the dynamics of trajectories and the cell population ratios. The balanced OT, formed by removing the growth term *g* from TIGON, fails to predict the stationary quiescent cells, and consequently, a false transition is observed to compensate for changes in cell population (Fig. 2g and Supplementary Fig. 1c). On the other hand, two other balanced OT-based models, TrajectoryNet^25^ and MIOFlow^26^, successfully circumvent the false transition by employing different objective functions and additional regularization terms (Supplementary Fig. 1d,e). However, their computed velocity shows disorganized directions with large magnitudes in the quiescent state or the late stage of transition state. Moreover, they show a relatively unchanged population ratio, which is inconsistent with the ground truth (Supplementary Fig. 1c). Overall, TIGON achieves better accuracy in predicting velocity and the ratio of cell population between two groups (Fig. 2h,i), whereas TrajectoryNet has better accuracy in trajectory prediction (Supplementary Fig. 1f and Supplementary Notes 2 and 3). Furthermore, we made comparisons with single-cell pseudotime methods through the standard metrics^9^ used in the benchmark (Supplementary Fig. 1g and Supplementary Notes 2 and 3).

Finally, we compared GRNs inferred between TIGON and 12 other GRN inference methods (Supplementary Notes 2 and 3). Specifically, the 11 methods implemented in BEELINE^37^ and CellOracle^38^ were included for comparison. Among these 13 methods, TIGON and CellOracle are the only two methods that consider both causal effects and cell-type specific GRNs, while TIGON allows more complete network architecture, including the self-regulation for a gene (Fig. 2j, Supplementary Fig. 3 and Supplementary Table 1). Together, we found that in both the area under the precision-recall curve and the receiver operating characteristic curve (AUROC), two metrics used in BEELINE benchmark for classifying directed edges in GRN, TIGON has the highest values. In addition, TIGON achieves the second and third highest value in the Pearson and Spearman correlations, respectively, in predicting the weights of GRN’s edges with directions and signs.

### Model predictions align with lineage tracing experiments

We applied TIGON to a temporal scRNA-seq dataset in mouse hematopoiesis using a lineage tracing technique^39^. This dataset uses additional barcodes to track clones over time where cells at the same clone are descendants of the same progenitor cell at day 0, providing information for trajectories and growth of cells (Methods).

**Fig. 3:**
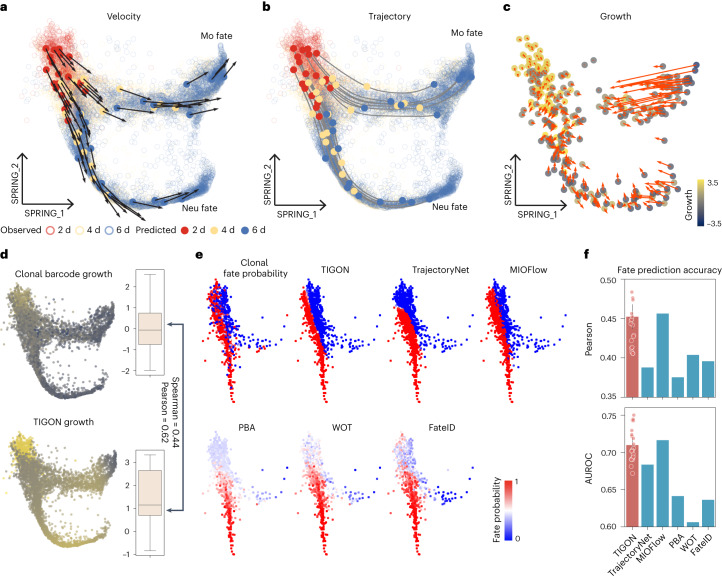
TIGON’s performance on the lineage tracing dataset. **a**–**c**, The data is visualized in force-directed layouts (SPRING plots). Cellular dynamics inference for velocity (**a**), trajectory (**b**) and growth and gradient of growth (**c**). **a**,**b**, Solid dots are cells predicted by TIGON where 20 cells were initially sampled from the density at day 2 and their snapshots at three time points are shown in different colours. The circles denote all observed cells from the scRNA-seq data. **c**, A total of 100 cells randomly sampled from densities at each time point for days 2, 4 and 6 are shown. **d**, Comparison between values of growth at day 2 and day 4 inferred by clonal barcode and TIGON in SPRING plots. Boxplots show distributions of growth for 5,210 cells in a five-number summary, where the centre line shows the median, the upper and lower limits of the box which show the IQR, spanning from the 25th to the 75th percentiles, and upper and lower whiskers show the maximum and the minimum. **e**, Fate probability for Neu fate estimations for day 2 cells using different methods that are listed from left to right in two rows: clonal fate probability from lineage barcode, TIGON, TrajectoryNet, MIOFlow, population balance analysis (PBA), Waddington-OT (WOT) and FateID. The clonal fate probability is taken as the ground truth for comparison. **f**, Barplots show accuracy in predicting clonal fate probability using (top) Pearson correlation and (bottom) the AUROC. The error bar for TIGON shows one standard deviation above and below the mean from *n* = 21 independent repeats. Scatter plots show the accuracy from each repeat.

Following the original study^39^, we pick cells in clones committing to neutrophils (Neu) and monocytes (M) fates at day 2, 4 and 6 (Fig. 3a). The data was first projected to the reduced two force-directed layouts (SPRING plots) after batch correction among different experiments. A bifurcation is clearly observed where early stage progenitor cells differentiate into Neu and Mo fates (Fig. 3a). Regarding the differentiation tendency, the reconstructed instantaneous cell transition velocity shows that bifurcation potentials are already detected at the early stage (day 2), becoming stronger at the later stages. At the final stage (day 6), the majority of cells commit their fates while continuing to move toward the distal end in each bifurcation branch. The trajectory analysis further demonstrates the bifurcation by tracking differentiation of each cell (Fig. 3b). To examine the predicted growth (Fig. 3c), we used shared clonal lineage barcodes to construct the ground truth growth for comparison (Fig. 3d, Methods and Supplementary Note 2). The Spearman and Pearson correlation between inferred growth and ground truth growth have the values of 0.44 and 0.62, respectively.

Next, we compared TIGON with other trajectory inference methods. The lineage tracing data tracks trajectories for clones of cells, while the computational methods infer trajectories for individual cells. Thus, the experiments cannot directly provide ground truth for computed trajectories. Instead we compared the fate probabilities for each cell between experiment and computations. We calculated the experimental clonal fate probability for each clone at day 2, based on the proportion of their descendant cells committing to Neu fate for that clone^39^. Similarly, the fate probability, defined on each cell at day 2 from computational methods, is the proportion of its descendant cells committed to Neu fate (Supplementary Note 2).

The clonal fate probabilities exhibit binary-like behaviours for cells at day 2 (Fig. 3e). The fate probability from TIGON shows a similar pattern with clonal fate probability. Unlike clonal fate probability, cells with two distinct fates predicted by TIGON are well-separated. Such binary-like fate probability is also captured by TrajectoryNet^25^ and MIOFlow^26^. In contrast, three approaches used in the original study^39^: population balance analysis (PBA)^13^, Waddington-OT (WOT)^23^ and FateID^40^, all fail to capture such binary-like behaviour. In particular cells away from the branching point show uncertain fates with fate probability around 0.5 (Fig. 3e). TIGON and MIOFlow show at least a 5% higher Pearson correlation with the ground truth clonal fate probability than those three approaches, and at least a 7% higher AUROC in fate classification with a threshold 0.5 (Fig. 3f).

### Reconstructing cellular dynamics in EMT

**Fig. 4:**
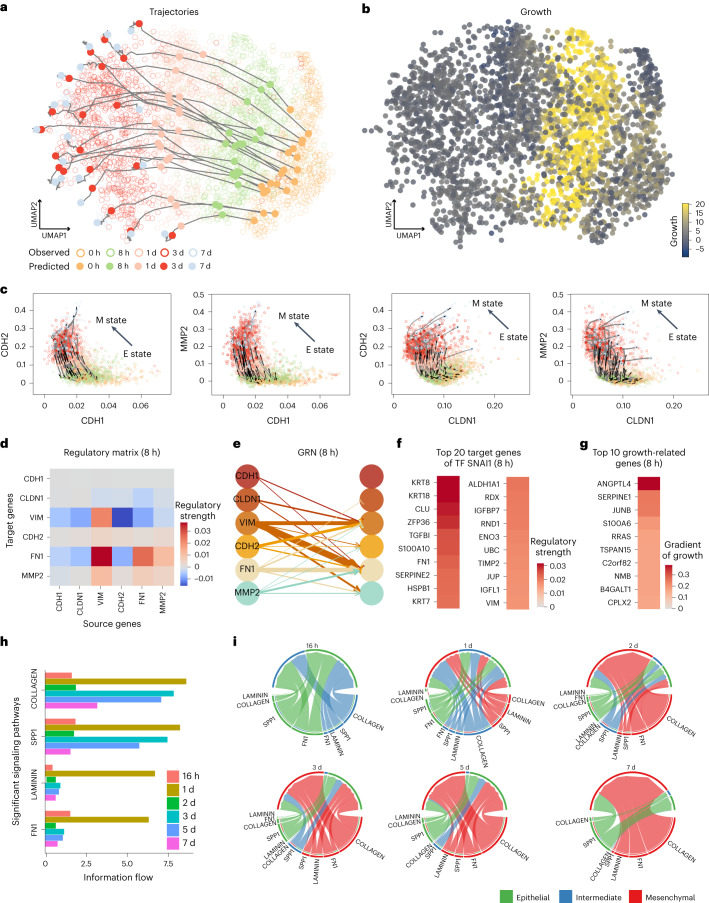
TIGON’s performance on the EMT scRNA-seq dataset. Results were obtained from a ten-dimensional latent space from AE. **a**,**b**, Visualization of TIGON’s outputs on UMAP space. **a**, Trajectories of 20 cells that are initially sampled from the density at 0 h, where solid dots show their snapshots at five time points. Circles show the observed cells from the scRNA-seq data. **b**, Values of growth for all observed cells. **c**, Trajectories and velocity for cells at gene expression space. Identical cells in **a** are shown in **c**. **d**,**e**, Regulatory matrix (**d**) and GRN (**e**) for six EMT marker genes for cells at 8 h. **f**, Regulatory matrix for top 20 target genes of an EMT TF SNAI1 for cells at 8 h. **g**, Gradient of growth for top ten growth-related genes for cells at 8 h. **h**, Barplots of information flow for the four signalling pathways with highest information flow inferred by CellChat. **i**, Chord diagrams from CellChat for cell–cell communications between epithelial, intermediate and mesenchymal cells at different time points. The inner thinner bar colours represent the targets that receive signal from the corresponding outer bar. The inner bar size is proportional to the signal strength received by the targets.

We next applied TIGON to a time-series scRNA-seq dataset from an A549 cancer cell line, in which cells were exposed to TGFB1 to induce EMT at the first five time points^41^. Cells collected at different time points were cultured in vitro with the identical initial cell numbers so that the numbers of cells collected at different time points directly represent the dynamics of cell population. We trained an AE with a ten-dimensional latent space, and used the latent space as the input for TIGON. To visualize outputs, we further projected the ten-dimensional latent space to two-dimensional UMAP. The time-series data indicates the early stage epithelial cells differentiate into intermediate state and then the final mesenchymal state (Fig. 4a). The inferred trajectories show similar transition dynamics. The reconstructed gene expression space from the latent space via AE shows decreasing expression level for two epithelial (E) markers (CDH1 and CLDN1) and increasing values for four mesenchymal (M) markers (VIM, CDH2, FN1 and MMP2) over time, indicating that TIGON can reconstruct dynamic gene expression (Fig. 4c, Supplementary Fig. 4a and Supplementary Note 1). Moreover, the patterns of TIGON-inferred growth exhibit higher values at the intermediate stage compared to the epithelial (E) or mesenchymal (M) stage (Fig. 4b), aligning with the previously reported strong stemness in intermediate stage cells^42,43^.

We further study GRNs involving in those two E markers and four M markers. Inhibitions from E markers to M markers were observed, especially to VIM and FN1 (Fig. 4d,e and Supplementary Fig. 4b,c). To study the temporal causal effects of transcription factors (TFs) on their target genes, we found that SNAI1, a canonical TF in EMT, exhibits positive regulation on VIM and FN1 (Fig. 4f). This finding aligns with the results of a previous study^44^. More potential target genes of SNAI1 were predicted from our GRN analysis (Fig. 4f and Supplementary Fig. 4d). To study the growth-related genes (Fig. 4g), we found that five out of the top ten growth-related genes are involved in cell growth reported in the UniProtKB database^45^. Specifically, they are ANGPTL4, JUNB, C2orf82, NMB and B4GALT1. Interestingly, B4GALT1 has been reported to be involved in epithelial cell proliferation^45^.

The inferred cellular dynamics provide single-cell gene expression levels at the unmeasured time points (Methods). Here we used CellChat^46^ to explore the cell–cell communication changes between E, M and intermediate state over time. At day 1, there is a noticeable upregulation in the COLLAGEN, FN1, SPP1 and LAMININ signalling pathways (Fig. 4h,i). An interesting observation is that COLLAGEN and SPP1 are downregulated at day 2, followed by upregulation at day 3, which contrasts with the downward trend perceived when considering the original measurement points (days 1, 3 and 7). Specifically, the COLLAGEN outgoing strength from the intermediate state decreases on day 2, then its outgoing strength from the M state increases on day 3 (Supplementary Fig. 5), while SPP1 follows a similar trend. Those cellular communication results require TIGON’s ability to reconstruct information at the unmeasured time points.

To study consistency across different dimension reduction methods, we analysed PCA and AE using two to ten dimensions for TIGON (Supplementary Figs. 6–9 and Supplementary Note 4). In higher dimensions, the computed velocity shows consistent direction, with the value of the cosine similarity greater than or around 0.5 (Supplementary Fig. 7a). As the dimensionality increases, the mean squared error (m.s.e.) for velocity between two different dimensions using the same dimension reduction method diminishes, suggestive of higher consistency (Supplementary Fig. 7b). We then calculated the Pearson correlation of the inferred growth between every pair of different dimension reductions, which yields values around 0.5 or higher (Supplementary Fig. 7c). The Pearson correlation of GRNs remains positive across all dimension reduction methods, exceeding or hovering around 0.5 when the dimension is greater than two (Supplementary Fig. 7d). For the gradient of growth, the correlation is similarly above 0.5 for dimensions greater than two (Supplementary Fig. 7e). Taken together, TIGON yields relatively consistent results across different dimension reductions and a wide range of latent space dimensions.

**Fig. 5:**
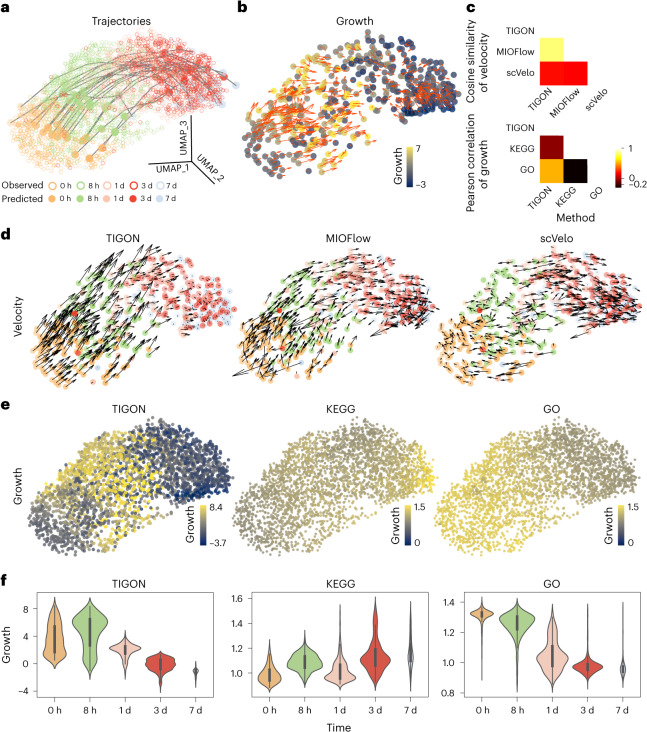
Comparisons of TIGON with trajectory inference or growth inference methods on the EMT scRNA-seq dataset. Results were obtained from three-dimensional UMAP space. **a**,**b**, Visualization of TIGON’s outputs. **a**, Trajectories of 20 cells that are initially sampled from the density at 0 h, where solid dots show their snapshots at five time points. Circles show the observed cells from the scRNA-seq data. **b**, Values of growth. At each time point, 100 cells are randomly sampled from the density. **c**, Comparisons of (top) inferred velocity and (bottom) growth. **d**, Velocity for all observed cells from the scRNA-seq data inferred by (left) TIGON, (middle) MIOFlow and (right) scVelo. **e**, Values of growth for all observed cells inferred by (left) TIGON, (middle) KEGG and (right) GO. **f**, Violin plots for inferred values of growth at different time points: (left) TIGON, (middle) KEGG and (right) GO. The width of the violin plot corresponds to the density of the data, showing a visual representation of the distribution at different growth values. Inside each violin, the white dot shows the median. The thick central bar of the box plot represents the IQR, spanning from the 25th to the 75th percentiles. The thin grey whiskers extend from the IQR to the maximum and minimum values within 1.5 times the IQR. Sample sizes for each time point from day 0 to day 7 are 577, 885, 788, 754 and 129, respectively.

Last, we compared TIGON with two other trajectory inference methods, MIOFlow^26^ and scVelo^12^. Unlike the velocity inferred from TIGON and MIOFlow, the velocity learned from scVelo seems to show unorganized directions, inconsistent with the temporal transition patterns (Fig. 5a–d). While using KEGG annotations of cell cycle and apoptosis genes which were also highly variable in the dataset to estimate growth, an approach described in a previous work^18^, the cells at the final stage achieve highest potential to divide (Fig. 5e,f and Supplementary Note 2). It is different from the experimental observations where smaller numbers of cells were observed at day 7 indicating low dividing potential (Fig. 5e,f)^41^. Using cell cycle and apoptosis genes from GO draws opposite conclusions to KEGG: the growth decreases during EMT. The inferred growth from the GO gene list may better fit with experimental observations. Nonetheless, the estimation of growth from genes highly depends on prior knowledge (for example, gene sets). Without prior knowledge of cell cycle and apoptosis genes, TIGON provides an unbiased approach in learning cell transition and growth.

### Identifying bifurcation of directed differentiation in iPSCs

**Fig. 6:**
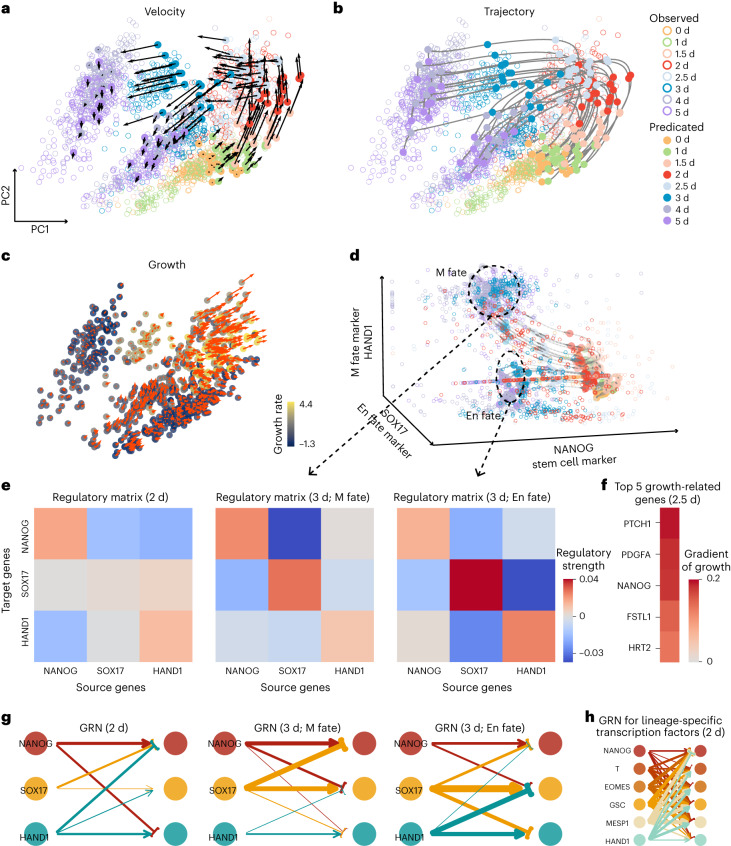
TIGON’s performance on the single-cell qPCR iPSC dataset with bifurcation. **a**–**c**, Visualization of TIGON’s outputs on first two PCs where TIGON was applied to the first four PCs. **a**,**b**, Velocity (**a**) and trajectories (**b**) of 20 cells initially sampled from the density at day 0, where solid dots show their snapshots at eight time points. Circles show observed cells from the data. **c**, Values of growth and gradient of growth. At each time point, 100 cells are randomly sampled from the density. **d**, Trajectories of cells on gene expression space of three bifurcation marker genes. **e**, Regulatory metrices for the three marker genes for cells at (left) day 2, (middle) day 3 with M fate and (right) day 3 with En fate. **f**, Gradient of growth for top five growth-related genes for cells at day 2.5. **g**, GRNs for the three marker genes for cells at (left) day 2, (middle) day 3 with M fate and (right) day 3 with En fate. **h**, GRN for lineage-specific transcription factors for cells at day 2.

Finally, we studied single-cell qPCR datasets at eight time points, showing a bifurcation process for differentiation of iPSCs in cardiomyocytes^47^ (Fig. 6a,b). The lineage-branching emerges after day 3 suggesting a bifurcation from a progenitor state to either a mesodermal (M) state or an endodermal (En) state.

TIGON reconstructs the instantaneous cell velocity and transition trajectory in driving the bifurcation process (Fig. 6a,b). At the early stages, cells have similar directions of velocity, but the heterogeneity increases over time. At the branching time (day 3), cells show two distinct directions of velocity, and they are segregated into M and En fates, respectively. During differentiation the two groups of cells remain well-separated. Large values of growth were observed near the branching time from day 2 to day 3 (Fig. 6c), suggesting a strong dividing potential at this point.

In this system, NANOG, SOX17 and HAND1 are marker genes for stem cells, En state and M state, respectively (Fig. 6d). The inferred GRNs in the three cell types consistently indicate self-activation for all three marker genes and mutual inhibitions between any pair of these marker genes (Fig. 6e–h and Supplementary Fig. 10). Interestingly, the toggle-switch interaction between HAND1 and SOX17, self-activation and mutual inhibition between two genes, was previously reported^47^. We then analysed the contribution of genes to the growth (Fig. 6f). The top five candidates at day 2 are all previously reported as growth-related genes in the UniProtKB database^45^. Specifically, PTCH1 is in a pathway playing a role in cell growth^48^, PDGRA and FSTL1 are growth factors^49,50^, NANOG is a TF involved in embryonic stem cell proliferation^51^ and HRT2 promotes the cell growth^52^.

To study the effect of dimension reductions, we further performed TIGON on the top eight principal components (PCs) (Supplementary Fig. 11). We found an ‘elbow’ around eight for the explained variances ratio versus PCs, suggesting that the majority of the information is captured using the first eight PCs (Supplementary Fig. 6f). Similar to the study in four PCs, the bifurcation takes place after day 3 and the largest growth is observed near the branching time from day 2 to day 3. The inferred growth yields a Pearson correlation of around 0.6 or higher (Supplementary Fig. 12e). The three marker genes indicate self-activation and mutual inhibition to each other (Supplementary Fig. 11d,e), suggesting consistent inference of GRNs. The top growth-related genes, such as NOTCH1 and FGF12, are known to be linked to growth, even though their order in the list is different from the study of four PCs.

## Discussion

TIGON is a deep learning method for extracting dynamical and gene mechanistic information from time-series single-cell transcriptomics data, allowing the coupling between the velocity of gene expression for each cell and the population growth. The dynamic unbalanced OT based on WFR distance shows promise for integrating temporal datasets while capturing cell division and death. As an efficient high-dimensional mesh-free deep learning method for the OT problem, TIGON may have other applications such as time-series single-cell ATAC-seq data or spatial transcriptome data. The computational methods in TIGON for solving dynamic unbalanced OT can be also applied to other areas such as image-image translation^53,54^.

Using reversible dimension reduction is important for TIGON to analyse dynamics of individual genes and their GRNs. Three methods, including AE, PCA and reversible UMAP^55^ (Supplementary Fig. 12 and Supplementary Notes 4 and 5), have been examined for various datasets. For gene analysis, reversible and differentiable dimensional reductions are required, such as AE and PCA. The dimension of the latent space usually needs to be greater than two to ensure the accuracy and reliability of results. The ‘elbow’ plot in PCA or a similar plot for reconstruction errors in AE are useful tools to determine an appropriate dimension (Supplementary Fig. 6).

The objective function in TIGON combines reconstruction errors and the cost function in WFR distance^31,32^. TIGON presents an optimal approach by combining short and long-term reconstruction errors to reduce the integration errors at different temporal scales (Supplementary Fig. 13 and Supplementary Note 6). Furthermore, different weights between Wasserstein and Fisher–Rao in WFR can produce consistent outputs ensuring the robustness of TIGON (Supplementary Figs. 14 and Supplementary Note 7).

Traditional mesh-based methods suffer from the curse of dimensionality in solving the high-dimensional dynamic OT problem. For a uniform spatial mesh with *N* grids at each dimension, the *O*(*N*^*d*^) calculation is needed for a *d*-dimensional system, which is often infeasible for high dimensions. Deep learning frameworks provide an efficient solution to such high-dimensional systems^56^. In this work, we have shown that the dimensionless solver in TIGON can directly solve the OT problem in ten dimensions. Solving a higher-dimensional problem (for example, 10^3^–10^4^ dimensions) will likely lead to additional computational challenges, such as stiffness in ODEs and large memory requirements. Development of efficient, stable and accurate numerical solvers^57^ coupled with memory-efficient neural ODEs^35^ methods may be critical.

Different experiments or techniques in transcriptomics data collection often lead to batch effects for different samples. We either used the low-dimensional representation from the original study where the batch correction has been performed or applied the Seurat protocol to remove batch effects. Since TIGON requires dimension reduction as a preprocessing step, a robust low-dimensional representation of the data is critical. In addition, TIGON requires adequate numbers of cells and time points (Supplementary Fig. 15 and Supplementary Note 8). Moreover, a small variance for the initial cell density is necessary to capture gene expression or low-dimensional space (Supplementary Fig. 16).

Cell populations may change over time due to cell division and death, which is important to include in the dynamical modelling of scRNA-seq data^13,58^. TIGON provides a fully unbiased approach to infer growth without the need for a preselected list of growth genes. The positive cosine similarity between velocity and gradient of growth in the transition cells in our study shows important synergy between growth and transition that needs to be considered in the model (Supplementary Fig. 17 and Supplementary Note 9).

Prior knowledge may be further considered to improve model accuracy^25^. For example, cells with different levels of potency may be identified by cell annotation to incorporate growth heterogeneity using different regularizations. RNA velocity may be added to constrain the transition velocity, and unspliced counts information may be useful to regularize the gene regulatory functions^59^. While TIGON reconstructs velocity and growth simultaneously, other important factors, such as signals from the microenvironment and communication among cells, may be important to include. Direct incorporation of cell–cell communication in the model remains challenging, particularly, for a large number of interactive cells in the high-dimensional gene expression space^60,61^. Applications of cell–cell communication inference methods, such as CellChat^46^ or exFINDER^62^, to single-cell gene expression inferred at unmeasured time points by TIGON, can produce dynamic cell–cell communication networks. For example, some cell–cell communication links may be similar at the measured time points; however, substantial changes take place between those points due to gene dynamics involved in such communications. Overall, TIGON provides an effective framework to connect temporal measurements for predicting novel dynamics that may not be seen directly from the data.

## Methods

### Data preprocessing

To efficiently use TIGON, for lineage tracing^39^, EMT^41^ and iPSC^47^ datasets, data was first projected to a low-dimensional space and taken as input for the TIGON method. We adopted the reduced two force-directed layouts (SPRING) space for lineage tracing data with batch correction^39^. For the EMT and iPSC datasets, four dimension reduction methods, including PCA, UMAP, reversible UMAP^55^ and AE, were employed. Specifically, AE was implemented in Pytorch packages^63^ and the other three methods were implemented using Seurat packages^64^. After obtaining the data at the low-dimensional space, each axis of the reduced space was scaled to [−2,2]. We have shown that the dimensionless solver in TIGON is capable to directly solve the OT problem around ten dimensions.

The time-series data was preprocessed before dimension reduction. For the EMT dataset, we obtained the processed Seurat (v.3)^64^ object from the original paper^41^. In the Seurat object, the data has been scaled and regressed out the potential batch effects from different experiments. For iPSC dataset, we obtained the log_2_Ex values from the original work with batch correction. Next, the top 3,000 highly variable genes in EMT were kept, and all 96 genes in iPSCs were used. When applying PCA, the log-transformed matrix was standardized such that each gene has zero mean and unit variance over all cells. UMAP and reversible UMAP used top 30 PCs. AE takes the log-transformed matrix without standardization, and details of its architecture and training procedure are discussed in Supplementary Note 1.

For lineage tracing dataset, we followed the original computational work to pick cells in clones committing to neutrophils (Neu) and monocytes (M) fates at days 2, 4 and 6 (ref. ^39^). For the EMT dataset, we picked data at the first five time points that are exposed to TGFB1 to induce EMT^41^. For the iPSC dataset^47^, data at all eight time points were used.

### Reconstruction of cell density

We first reconstructed cell densities generated from the time-series data within a *d*-dimensional space, using either the original gene expression space or the low-dimensional space obtained from dimension reduction. Suppose the time-series discrete data are given by

3 $$\left({t}_{1},{C}^{1}\right),\left({t}_{2},{C}^{2}\right),\cdots ,\left({t}_{T},{C}^{T}\right)$$

where ${C}^{i}={\left\{{c}_{{t}_{i}}^{\left(\,\,j\right)}\right\}}_{j=1}^{{N}^{i}}\in {{\mathbb{R}}}^{{N}^{i}\times d}$ is a set of *N*^*i*^ independent and identically distributed samples drawn from the distribution at a *d*-dimensional space at time *t*_*i*_. If no prior information about the mass is given, the number of samples *N*^*i*^ is proportional to the relative cell population. Here, we assume that when calculating the relative cell population changes over time, the variability introduced by sequencing techniques, such as cells not successfully sequenced, is negligible. We generated the density ${\rho }_{{t}_{i}}$ using a Gaussian mixture model that combines *N*^*i*^ Gaussian distributions with identical weights, each corresponding to a sample point. Each of these distributions has its mean at a corresponding sample point and a covariance matrix that is a scaled identity matrix $\varSigma ={\sigma} I\in {{\mathbb{R}}}^{d\times d}$ with a constant standard deviation *σ* for all sample points. The density ${\rho }_{{t}_{i}}$ is then obtained by the mixture Gaussian distribution multiplied by the relative population with respect to initial time point *t*_1_, which is ${\widetilde{N}}^{i}$.

For the lineage tracing dataset, one set of initial cells were cultured, and one portion of all remaining cells were collected for sequence at each time point. They are 50%, 30% and 100% for day 2, day 4 and day 6, in the experiment, respectively. The relative cell population is ${\widetilde{N}}^{1}=1$, ${\widetilde{N}}^{2}=\frac{{N}^{2}/30 \% }{{N}^{1}}$ and ${\widetilde{N}}^{3}=\frac{{N}^{3}/70 \% }{{N}^{1}}$, for day 2, day 4 and day 6, respectively.

For the EMT dataset, samples collected at different time points were cultured from the identical initial number of cells. In this case, the numbers of samples at different time points are directly proportional to the total cell population. Similar to the iPSC dataset, the relative cell population is defined as ${\widetilde{N}}^{1}=\frac{{N}^{1}}{{N}^{1}}=1$, ${\widetilde{N}}^{2}=\frac{{N}^{2}}{{N}^{1}}$, …, ${\widetilde{N}}^{T}=\frac{{N}^{T}}{{N}^{1}}$.

### Dynamic optimal transport

This section presents brief reviews of dynamic OT introduced by Benamou and Brenier^24^. This framework models the transport in a continuum sense utilizing the fluid dynamic framework. Suppose the data is subject to a smooth and time-dependent density *ρ*(*x,t*) ≥ 0, the spatial-temporal dynamics of the density is governed by the continuity equation

4 $${\partial }_{t}\rho +\nabla \cdot \left(\mathbf{v} \rho \right)=0$$

for all *t* ∈ [0,*T*], $x\in {{\mathbb{R}}}^{d}$ and the initial and final conditions:

5 $$\rho \left(\cdot ,0\right)={\rho }_{0},\rho \left(\cdot ,T\,\right)={\rho }_{T}$$

where $\mathbf{v}\left(x,t\right)\in {{\mathbb{R}}}^{d}$ describes the velocity field of the density movement. The transport map from the initial to final conditions is not unique, and OT adds a transport cost function being minimized to further constrain the optimization problem.

Considering the transport cost function between two points as the squared Euclidean distance, *c*(*x,y*) = |*x*−*y*|^2^, the cost function for dynamic OT is:

6 $$T\mathop{\int }\limits_{0}^{T}\mathop{\int }\limits_{{{\mathbb{R}}}^{d}}{\left|\mathbf{v}\left(x,t\right)\right|}^{2}\rho \left(x,t\right){{\mathrm{d}}x}{{\mathrm{d}}t}.$$

The minimized cost function is equivalent to Wasserstein distance in the case with *p* = 2 (ref. ^24^).

### Dynamic unbalanced optimal transport

A major constraint of dynamic OT is the assumption of the unchanged total mass. The mass conservation is not an appropriate approach in modelling biological systems for population distributions that involve birth (mass creation) and death (mass destruction). The unbalanced OT is increasingly used for connecting a time-series of densities with different mass. It introduces a growth term $g\left(x,t\right):{{\mathbb{R}}}^{d}\times \left[0,T\,\right]{\mathbb{\to }}{\mathbb{R}}$ to the continuity (equation (4)):

7 $$\begin{array}{ll} {\partial }_{t}\rho +\nabla \cdot \left({\bf {v}}\rho \right)=g\rho, \\ \rho \left(\cdot ,0\right)={\rho }_{0},\rho \left(\cdot ,T\,\right)={\rho }_{T} \end{array}$$

WFR distance^31,32^ has been used to constrain the transport dynamics with respect to both kinetic and growth energy. It minimizes the combination of quadratic Wasserstein and Fisher–Rao metrics simultaneously. The function being minimized for WFR distance in period [0,*T*] is:

8 $${W}_{0,T}=T\displaystyle\mathop{\int }\limits_{0}^{T}\mathop{\int }\limits_{{{\mathbb{R}}}^{d}}\left({\left|{\bf {v}}\left(x,t\right)\right|}^{2}+\alpha {\left|g\left(x,t\right)\right|}^{2}\right)\rho \left(x,t\right){{\mathrm{d}}x}{{\mathrm{d}}t},$$

where the minimum of $T{\int }_{\!0}^{T}{\int }_{{{\mathbb{R}}}^{d}}{\left|\mathbf{v}\left(x,t\right)\right|}^{2}\rho \left(x,t\right){{\mathrm{d}}x}{{\mathrm{d}}t}$ refers to the square of Wasserstein metric, and the minimum of $T{\int }_{\!0}^{T}{\int }_{{{\mathbb{R}}}^{d}}{\left|g\left(x,t\right)\right|}^{2}\rho \left(x,t\right){{\mathrm{d}}x}{{\mathrm{d}}t}$ refers to the square of Fisher–Rao metric. *α* is a hyperparameter to balance the effects of transport and growth explicitly, that is between quadratic Wasserstein and Fisher–Rao metrics. *α* = 1 was mainly examined in this work. Different values of *α* lead to consistent behaviours (Supplementary Fig. 14 and Supplementary Note 7).

## Dimensionless formulation

Numerical solvers may become computationally inefficient for high-dimensional problems. TIGON provides a dimensionless formulation for the high-dimensional dynamic unbalanced OT (equation (7)) with its cost function (equation (8)).

### Continuity equation with growth term

We first converted the high-dimensional continuity equation with the growth term into a system of ODEs based on a set of sample points outlined in the Lemma. The dynamics of density is then decomposed into each sample point along its trajectory *x*(*t*).

**Lemma**: If density $\rho \left(x,t\right):{{\mathbb{R}}}^{d}\times \left[0,T\,\right]\to {{\mathbb{R}}}^{+}$, velocity field $\mathbf{v}\left(x,t\right):{{\mathbb{R}}}^{d}\times \left[0,T\,\right]\to {{\mathbb{R}}}^{d}$ and growth $g\left(x,t\right):{{\mathbb{R}}}^{d}\times \left[0,T\,\right]{\mathbb{\to }}{\mathbb{R}}$ satisfy

$$\left\{\begin{array}{c}{\partial }_{t}\rho \left(x,t\right)+\nabla \cdot \left(\mathbf{v}\left(x,t\right)\rho \left(x,t\right)\right)=g\left(x,t\right)\rho \left(x,t\right)\\ \rho \left(x,0\right)={\rho }_{0}\left(x\right)\end{array}\right.$$

for all 0 ≤ *t* ≤ *T* where $$\left\{\begin{array}{c}\frac{{{\mathrm{d}}x}\left(t\right)}{{{\mathrm{d}}t}}=\mathbf{v}\left(x,t\right)\\ x\left(0\right)={x}_{0}\end{array}\right.$$, then we have $\frac{{\mathrm{d}}\left(\mathrm{ln}\rho \right)}{{{\mathrm{d}}t}}=g-\nabla \cdot \mathbf{v}$.

Proof:

$$\frac{\partial \rho }{\partial t}=g\rho -\nabla \cdot \left(\mathbf{v}\rho \right)=g\rho -\nabla \rho \cdot \mathbf{v}-\rho \nabla \cdot \mathbf{v}$$

$$\begin{array}{lll}\frac{{\mathrm{d}}\rho }{{{\mathrm{d}}t}} &=&\nabla \rho \cdot \dfrac{{{\mathrm{d}}x}}{{{\mathrm{d}}t}}+\dfrac{\partial \rho }{\partial t}\\ &=&\nabla \rho \cdot \mathbf{v}+\dfrac{\partial \rho }{\partial t}\\ &=&\nabla \rho \cdot \mathbf{v}+g\rho -\nabla \rho \cdot \mathbf{v}-\rho \nabla \cdot \mathbf{v}\\ &=&g\rho -\rho \nabla \cdot \mathbf{v}\end{array}$$

So that $$\frac{{\mathrm{d}}\left(\mathrm{ln}\rho \right)}{{{\mathrm{d}}t}}=g-\nabla \cdot \mathbf{v}$$

### Cost function in WFR

Then, we derived an equivalent dimensionless form of the cost function in WFR metric:

9 $$\begin{array}{ccc}{W}_{0,T} & = &\displaystyle T\mathop{\int }\limits_{0}^{T}\mathop{\int }\limits_{{{\mathbb{R}}}^{d}}\left({\left|\mathbf{v}\left(x,t\right)\right|}^{2}+\alpha {\left|g\left(x,t\right)\right|}^{2}\right)\rho \left(x,t\right){{\mathrm{d}}x}{{\mathrm{d}}t}\\ & = &\displaystyle T{\,{\mathbb{E}}}_{{x}_{0} \sim {\rho }_{0}}\mathop{\int }\limits_{0}^{T}\left({\left|\mathbf{v}\left(x,t\right)\right|}^{2}+\alpha {\left|g\left(x,t\right)\right|}^{2}\right)\rho \left(x,t\right){{\mathrm{d}}t}\end{array}$$

where ${{\mathbb{E}}}_{{x}_{0} \sim {\rho }_{0}}\left[\cdot \right]$ denotes that the expectation for random variable *x*_0_ followed distribution *ρ*_0_. We assume the characteristic curves do not intersect, and the derivation is given in the theorem below:

**Theorem**: If smooth density $\rho \left(x,t\right):{{\mathbb{R}}}^{d}\times \left[0,T\,\right]\to {{\mathbb{R}}}^{+}$, velocity field $\mathbf{v}\left(x,t\right):{{\mathbb{R}}}^{d}\times \left[0,T\,\right]\to {{\mathbb{R}}}^{d}$ and growth rate $g\left(x,t\right):{{\mathbb{R}}}^{d}\times \left[0,T\,\right]{\mathbb{\to }}{\mathbb{R}}$ satisfy

$$\left\{\begin{array}{c}{\partial }_{t}\rho \left(x,t\right)+\nabla \cdot \left(\mathbf{v}\left(x,t\right)\rho \left(x,t\right)\right)=g\left(x,t\right)\rho \left(x,t\right)\\ \rho \left(x,0\right)={\rho }_{0}\left(x\right)\end{array}\right.$$

for all 0 ≤ *t* ≤ *T* where $$\left\{\begin{array}{c}\frac{{{\mathrm{d}}x}\left(t\right)}{{{\mathrm{d}}t}}=\mathbf{v}\left(x,t\right)\\ x\left(0\right)={x}_{0}\end{array}\right.$$, then for any measurable function $f\left(x,t\right):{{\mathbb{R}}}^{d}\times \left[0,T\,\right]\to {{\mathbb{R}}}^{d}$, we have

$$\mathop{\int }\limits_{0}^{{T}}\mathop{\int }\limits_{{{\mathbb{R}}}^{d}}f\left(x,t\right)\rho \left(x,t\right)\mathrm{d}x\mathrm{d}t={{\mathbb{E}}}_{{x}_{0}\sim {\rho }_{0}}\mathop{\int }\limits_{0}^{{T}}f\left(x,t\right){e}^{\mathop{\int }\nolimits_{0}^{{t}}g\left(x,s\right)ds}\mathrm{d}t.$$

Proof:

Let *σ*(*x*_0_,*t*) = *x*(*t*), then by Jacobi’s formula

$$\begin{array}{ccl}\frac{\partial }{\partial t}\left|\frac{\partial \sigma }{\partial {x}_{0}}\right| & = & {\rm{Tr}}\left({\rm{adj}}\left(\frac{\partial {\rm{\sigma }}}{\partial {x}_{0}}\right)\frac{\partial }{\partial t}\frac{\partial {\rm{\sigma }}}{\partial {x}_{0}}\right)\\ & = & {\rm{Tr}}\left({\rm{adj}}\left(\frac{\partial {\rm{\sigma }}}{\partial {x}_{0}}\right)\frac{\partial \left(\frac{\partial {\rm{\sigma }}}{\partial {\rm{t}}}\right)}{\partial {x}_{0}}\right)\\ & = & {\rm{Tr}}\left({\rm{adj}}\left(\frac{\partial {\rm{\sigma }}}{\partial {x}_{0}}\right)\frac{\partial \mathbf{v}}{\partial x}\frac{\partial {\rm{\sigma }}}{\partial {x}_{0}}\right)\\ & = & {\rm{Tr}}\left(\frac{\partial {\rm{\sigma }}}{\partial {x}_{0}}{\rm{adj}}\left(\frac{\partial {\rm{\sigma }}}{\partial {x}_{0}}\right)\nabla \mathbf{v}\right)\\ & = & \left|\frac{\partial \sigma }{\partial {x}_{0}}\right|\nabla \cdot \mathbf{v}\end{array}$$

The fourth equation utilizes the symmetric property of the trace: Tr(*AB*) *=* Tr(*BA*). The last equation utilizes the property of determinant: det(*A*)*I* *=* *A* adj(*A*). Then we have:

$$\begin{array}{ccl}\frac{{\mathrm{d}}}{{{\mathrm{d}}t}}\left(\rho \left|\frac{\partial \sigma }{\partial {x}_{0}}\right|\right) & = & \frac{{\mathrm{d}}\rho }{{{\mathrm{d}}t}}\left|\frac{\partial \sigma }{\partial {x}_{0}}\right|+\rho \frac{{\mathrm{d}}}{{{\mathrm{d}}t}}\left(\left|\frac{\partial \sigma }{\partial {x}_{0}}\right|\right)\\ & = & \left(\frac{\partial \rho }{\partial x}\mathbf{v}+\frac{\partial \rho }{\partial t}\right)\left|\frac{\partial \sigma }{\partial {x}_{0}}\right|+\rho \left|\frac{\partial \sigma }{\partial {x}_{0}}\right|\nabla \cdot \mathbf{v}\\ & = & \left(\nabla \rho \cdot \mathbf{v}+g\rho -\nabla \cdot \left(\mathbf{v}\rho \right)\right)\left|\frac{\partial \sigma }{\partial {x}_{0}}\right|+\rho \left|\frac{\partial \sigma }{\partial {x}_{0}}\right|\nabla \cdot \mathbf{v}\\ & = & g\rho \left|\frac{\partial \sigma }{\partial {x}_{0}}\right|\end{array}$$

Let $$\rho \left|\frac{\partial \sigma }{\partial {x}_{0}}\right|=M\left(t\right)$$, then $$M\left(t\right)=M\left(0\right){e}^{\mathop{\int }\nolimits_{0}^{t}g\left(x,s\right)\mathrm{d}s}$$

$$\begin{array}{ccl}\displaystyle\mathop{\int }\limits_{{{\mathbb{R}}}^{d}}f\left(y,t\right)\rho \left(y,t\right){\mathrm{d}y} & = & \displaystyle\mathop{\int }\limits_{{{\mathbb{R}}}^{d}}f\left(\sigma \left({x}_{0},t\right),t\right)\rho \left(\sigma \left({x}_{0},t\right),t\right)\left|\frac{\partial \sigma }{\partial {x}_{0}}\right|\mathrm{d}{x}_{0}\\ & = & \displaystyle\mathop{\int }\limits_{{{\mathbb{R}}}^{d}}f\left(\sigma \left({x}_{0},t\right),t\right)\rho \left(\sigma \left({x}_{0},0\right),0\right){e}^{\mathop{\int }\nolimits_{0}^{{t}}g\left(x,s\right){ds}}\mathrm{d}{x}_{0}\\ & = & {{\mathbb{E}}}_{{x}_{0}\sim {\rho }_{0}}\,f\left(x,t\right){e}^{\mathop{\int }\nolimits_{0}^{{t}}g\left(x,s\right)\mathrm{d}s}\end{array}$$

## Reconstruction errors

The Lemma allows the computation of density dynamics at each trajectory *x*(*t*) with an initial value. The model needs to minimize the reconstruction errors between the estimated density and the ground truth density. The reconstruction errors take the m.s.e. between the ground truth and the estimated density of a set of sample points at multiple time points.

To calculate the estimated density, a ground truth density at a different time point needs to be taken as the initial conditions. Without loss of the generality, we consider a pair of time points *t*_*i*_ < *t*_*j*_, where estimated density at *t*_*j*_ is obtained by integrating the ground truth density at *t*_*i*_ using the equivalent form of equation (7) in the Lemma. We picked a set of samples from the ground truth density at later time ${x}_{{t}_{j}}\sim {\rho }_{{t}_{j}}$, and integrated them backward to the early time point *t*_*i*_ along the trajectory:

10 $${\hat{x}}_{{t}_{i}}={x}_{{t}_{j}}+\mathop{\int }\limits_{{t}_{j}}^{{t}_{i}}\mathbf{v}\left(x,t\right){{\mathrm{d}}t}$$

The value of ground truth density for these samples at ${t}_{i}$, ${\rho }_{{t}_{i}}\left({\hat{x}}_{{t}_{i}}\right)$ is obtained. Then we integrated the density from these sample points forward to ${x}_{{t}_{j}}$. The value of the estimated density for the same initial samples was calculated:

11 $$\mathrm{ln}{\widetilde{\rho }}_{{t}_{j}}\left({x}_{{t}_{j}}\right)=\mathrm{ln}{\rho }_{{t}_{i}}\left({\hat{x}}_{{t}_{i}}\right)-\mathop{\int }\limits_{{t}_{j}}^{{t}_{i}}\frac{{\mathrm{d}}\mathrm{ln}\rho }{{{\mathrm{d}}t}}{{\mathrm{d}}t},$$

where ${\widetilde{\rho }}_{{t}_{j}}$ denotes the estimated density at *t*_*j*_. In equations (10) and (11), we integrated back and forth between *t*_*i*_ and *t*_*j*_, which follows the procedure for training normalizing flows^34^. This technique allows that the sample points ${x}_{{t}_{j}}=x\left({t}_{j}\right)$ follow the distribution of the ground truth. Suppose we have *K* samples, the reconstruction error is denoted as

12 $${R}_{{t}_{i},{t}_{j}}=\frac{1}{K}\mathop{\sum }\limits_{k=1}^{K}{\left[{\widetilde{\rho }}_{{t}_{j}}\left({x}_{{t}_{j}}^{\left(k\right)}\right)-{\rho }_{{t}_{j}}\left({x}_{{t}_{j}}^{\left(k\right)}\right)\right]}^{2}.$$

We consider short-term reconstruction error, ${R}_{{t}_{i},{t}_{i+1}}$, and long-term reconstruction error, ${R}_{{t}_{1},{t}_{i+1}}$. The combined reconstruction errors facilitate robust and accurate results by minimizing errors at different time scales (Supplementary Note 6). The combined reconstruction error includes both types of errors at different time points:

13 $$R=\mathop{\sum }\limits_{i=1}^{T-1}{R}_{{t}_{i},{t}_{i+1}}+\mathop{\sum }\limits_{i=1}^{T-1}{R}_{{t}_{1},{t}_{i+1}}$$

## Deep learning-based dimensionless solver in TIGON

Now we take everything together to derive the deep learning-based dimensionless solver for TIGON, including forward propagation via the ODE solver, and backward propagation through neural ODEs.

First, two fully connected neural networks are used to estimate velocity **v**(*x,t*) and growth rate *g*(*x,t*) in the continuity equation (equation (7)) where the input is a sample point *x* and time *t*.

Then the cost function in WFR metric is computed by summing up the cost between all pairs of consecutive time points:

14 $$W=\mathop{\sum }\limits_{i=1}^{T-1}{W}_{{t}_{i},{t}_{i+1}}.$$

Specifically, ${W}_{{t}_{i},{t}_{i+1}}$ is defined as the following:

15 $${W}_{{t}_{i},{t}_{i+1}}=\left({t}_{i+1}-{t}_{i}\right){{\mathbb{E}}}_{{x}_{i}\sim {\rho }_{{t}_{i}}}\mathop{\int }\limits_{{t}_{i}}^{{t}_{i+1}}\left({\left|\mathbf{v}\left(x,t\right)\right|}^{2}+\alpha {\left|g\left(x,t\right)\right|}^{2}\right){e\,}^{\mathop{\int }\nolimits_{{t}_{i}}^{t}g\left(x,s\right){\mathrm{d}s}}{\mathrm{d}t}$$

where *x* = *x*(*t*) is the trajectory satisfying

16 $$\left\{\begin{array}{c}\frac{{{\mathrm{d}}x}\left(t\right)}{{{\mathrm{d}}t}}=\mathbf{v}\left(x,t\right)\\ x\left({t}_{i}\right)={x}_{i}\end{array}\right.$$

The reconstruction error is computed using equation (13). Then the loss function is taken as the weighted sum of cost and reconstruction error:

17 $${\rm{Loss}}={{W}}+{{{\lambda }}}_{d}{R}$$

with hyperparameter *λ*_*d*_. In particular, the samples for computing loss are randomly selected every epoch during training to enhance the model robustness.

Temporal integral (equation (15)) and ODEs (equation (16)) were numerically solved by an ODE solver DOPRI5 method, a Runge–Kutta method with adaptive step size. The gradients of the loss function with respect to the parameters in two neural networks for **v**(*x,t*) and *g*(*x,t*) were computed by naive method in neural ODEs^34^ with a memory-efficient implementation^35^. The Adam optimizer was employed to update the gradient^65^.

The deep learning-framework in TIGON was implemented with a Pytorch package^63^. The two neural networks took the same architectures. Specifically, a fully connected layer is followed by a Tanh activation function, except the output layer, which has no activation function. The hyperparameters used for each dataset are summarized in Supplementary Table 2. The pseudocode of the workflow of the training process in our method is presented in the Supplementary Information pseudo code.

## Three-gene simulation model

In the three-gene simulation model, its GRN structure is described in Fig. 2a. This regulatory relationship is modelled by a system of stochastic ODEs:

18 $$\begin{array}{ccl}\frac{{{\mathrm{d}}A}}{{{\mathrm{d}}t}} & = & \frac{{C}_{A}{A}^{2}+S}{1+{C}_{A}{A}^{2}+{H}_{B}{B}^{2}+{H}_{C}{C}^{\,2}+S}-{d}_{A}A+{\hat{\sigma }}_{A}{\xi }_{t}\\ \frac{{{\mathrm{d}}B}}{{{\mathrm{d}}t}} & = & \frac{{C}_{B}{B}^{2}+S}{1+{H}_{A}{A}^{2}+{C}_{B}{B}^{2}+{H}_{C}{C}^{\,2}+S}-{d}_{B}B+{\hat{\sigma }}_{B}{\xi }_{t}\\ \frac{{{\mathrm{d}}C}}{{{\mathrm{d}}t}} & = & \frac{{C}_{C}{C}^{2}}{1+{C}_{C}{C}^{\,2}}-{d}_{C}C+{\hat{\sigma }}_{C}{\xi }_{t}\end{array}$$

The genes *A* and *B* mutually inhibit each other and have self-activation for their own expression, which form a toggle switch^66^. There is an external signal, *S*, that provides sources to activate both *A* and *B* with constant strengths that are independent of gene expression levels. Gene *C* strongly inhibits both *A* and *B* expression. *A*(*t*), *B*(*t*) and *C*(*t*) are a concentration of genes at time *t*. *C*_*A*_, *C*_*B*_ and *C*_*C*_ are strengths of self-activation for three genes, and *H*_*A*_, *H*_*B*_ and *H*_*C*_ are strengths of inhibition from *A*, *B* and *C*. The signal, inhibition and self-activation are modelled by hill functions. In addition, *d*_*A*_*A*, *d*_*B*_*B* and *d*_*C*_*C* are degradations for genes *A*, *B* and *C*, respectively. $\hat{{\rm{\sigma }}}{\xi }_{t}$ is the additive white noise for stochastic effects on gene expression. The probability of cell division is positively correlated with gene *B*: $g=\frac{{B}^{2}}{1+{B}^{2}} \%$. Every time a cell divides, two cells inherit the gene expression state of their parent cell, (*A*(*t*), *B*(*t*), *C*(*t*)), with independent perturbations ${\hat{\sigma }}_{d}{\mathscr{N}}\left(\mathrm{0,1}\right)$ on each gene, and make cell transition independently afterward.

In this work, we used one set of parameters: *C*_*A*_ = *H*_*A*_ = 0.5, *C*_*B*_ = *H*_*B*_ *=* *C*_*C*_ = 1, *H*_*C*_ = 10, *d*_*A*_ = *d*_*B*_ *=* *d*_*C*_ = 0.4, ${\hat{\sigma }}_{A}={\hat{\sigma }}_{B}=0.05$ and ${\hat{\sigma }}_{C}=0.01$, ${\hat{\sigma }}_{D}=0.014$. We generated two groups of initial cells which are independent and identically distributed from two normal distributions *N*([2,0.2,0],0.01) and *N*([0,0,2],0.01) in the three-dimensional gene space. The stochastic differential equation was solved by the Euler–Maruyama method using the time step Δ*t* = 0.2. At each time step, we corrected the negative expression to be 0. The training data for TIGON took data at time *t* = 0, 10, 20, 30 and 40, and the input densities were generated by a Gaussian mixture model with standard deviation *σ* = 0.2.

## Computations of RNA velocity

RNA velocity was calculated on the EMT dataset. We obtained the processed Seurat (v.3)^64^ object and the loom file with spliced and unspliced mRNA counts for each cell and gene from the original paper^41^. We followed the procedure recommended by scVelo to compute RNA velocity for the EMT dataset^12^. We selected the top 2,000 highly variable genes and normalized the mRNA counts within each cell using the function scv.pp.filter_and_normalize in scVelo. The first- and second-order moments were computed using the top 30 PCs and the top 30 nearest neighbours with the funciton scv.pp.moments. The RNA velocities were then computed using the function scv.tl.velocity function with mode = ‘dynamical’.

## Temporal cell–cell communications inference

For the EMT dataset, the generated data for temporal cell–cell communication inference was inferred from cellular dynamics learned on the ten-dimensional latent space of AE. Specifically, 1,000 cells were randomly sampled from initial density and the states of the cells at 16 h and days 1, 2, 3, 5 and 7 were further predicted via integration of inferred velocity. Those generated temporal data were then projected back to the 3,000 highly variable gene space via the decoder of AE.

We merged the cells from six time points into one Seurat object^64^. We then followed the procedure in Seurat to find the clustering. We scaled and ran PCA on the data using ScaleData and RunPCA in Seurat. The three clusters were then computed using the top ten PCs, top 150 nearest neighbours and 0.1 resolution with the functions FindNeighbors and FindClusters. The three clusters were annotated as epithelial, intermediate and mesenchymal states based on the time where the cluster including most of the cells from early time points was an epithelial state, the cluster including most of the cells from day 7 was a mesenchymal state and the remaining cluster was an intermediate state. CellChat^46^ was then performed to compute the temporal cell–cell communication among the three states.

### Reporting summary

Further information on the research design is available in the Nature Portfolio Reporting Summary linked to this article.

### Supplementary information

Supplementary Information Supplementary Figs. 1–17, Pseudo code, Notes 1–9 and Tables 1–3.

Reporting Summary

## Data Availability

Data for the single-cell lineage tracing was downloaded from https://github.com/AllonKleinLab/paper-data/tree/master/Lineage_tracing_on_transcriptional_landscapes_links_state_to_fate_during_differentiation (ref. ^39^). Data for TGFB1-induced EMT from A549 cancer cell line was downloaded from https://github.com/dpcook/emt_dynamics (ref. ^41^). Data for single-cell qPCR dataset of iPSCs toward cardiomyocytes was downloaded from https://www.ncbi.nlm.nih.gov/pmc/articles/PMC5338498/bin/pnas.1621412114.sd02.xlsx (ref. ^47^).

## Code availability

All source codes and models are publicly available at https://github.com/yutongo/TIGON (ref. ^67^).
