# Supplementary material for: Reconstructing growth and dynamic trajectories from single-cell transcriptomics data
Source: Nat Mach Intell. 2023 Nov 30;6(1):25–39. doi: 10.1038/s42256-023-00763-w (PMC10805654; doi:10.1038/s42256-023-00763-w)
Supplement: Supplementary file 1 — Supplementary Figs. 1–17, Pseudo code, Notes 1–9 and Tables 1–3. [file 42256_2023_763_MOESM1_ESM.pdf]

nature machine intelligence

Article https://doi.org/10.1038/s42256-023-00763-w

# Reconstructing growth and dynamic trajectories from single-cell transcriptomics data

In the format provided by the authors and unedited

**This file includes the following subsections:**


1. **Supplementary Figures:**

   - Supplementary Figure 1: Trajectory inference benchmark on simulated data.
   - Supplementary Figure 2: TIGON's performance on simulated data.
   - Supplementary Figure 3: Gene regulatory network inference benchmark on simulated data.
   - Supplementary Figure 4: TIGON's performance on EMT dataset using ten-dimensional autoencoder latent space.
   - Supplementary Figure 5: Temporal cell-cell communication from TIGON for EMT dataset.
   - Supplementary Figure 6: Information captured in dimension reduction methods with various dimensions.
   - Supplementary Figure 7: Consistency of TIGON's performance on different dimension reductions (DRs).
   - Supplementary Figure 8: TIGON's performance on EMT dataset using eight-dimensional autoencoder latent space.
   - Supplementary Figure 9: TIGON's performance on EMT dataset using first ten principal components (PCs).
   - Supplementary Figure 10: TIGON's performance on iPSCs dataset using first four principal components (PCs).
   - Supplementary Figure 11: TIGON's performance on iPSCs dataset using first eight principal components (PCs).
   - Supplementary Figure 12: TIGON's performance on iPSCs dataset using reversible UMAP.
   - Supplementary Figure 13: TIGON's performance without long-term reconstruction error for simulated dataset.
   - Supplementary Figure 14: TIGON's performance when changing the weight between Wasserstein and Fisher-Rao for simulated dataset.
   - Supplementary Figure 15: TIGON's performance using different number of samples and time points as input for simulated data.
   - Supplementary Figure 16: Morphology of data captured by Gaussian mixture model.
   - Supplementary Figure 17: Comparisons of directionality between velocity and gradient of growth.

2. **Pseudo code: TIGON algorithm**

3. **Supplementary Notes:**

   - Supplementary Note 1: Gene analysis utilizing reversible and differentiable dimension reduction methods

- Supplementary Note 2: Quantification and evaluating metrics
- Supplementary Note 3: Benchmarking TIGON against other trajectory and GRN inference methods on simulated data
- Supplementary Note 4: Impacts of dimension reductions on TIGON for EMT dataset
- Supplementary Note 5: TIGON using revsersible UMAP for iPSCs dataset
- Supplementary Note 6: Necessity of including long-term reconstruction error in the loss function
- Supplementary Note 7: Exploration of weights between Wasserstein and Fisher-Rao in Wasserstein-Fisher-Rao metric
- Supplementary Note 8: Impacts of number of samples and time points in training data
- Supplementary Note 9: Synergy between gradient of growth and velocity

4. **Supplementary Tables:**

- Supplementary Table 1: Summery of GRN inference methods.
- Supplementary Table 2: List of hyperparameters used in the training process.
- Supplementary Table 3: Autoencoder (AE) architecture and hyperparameters.

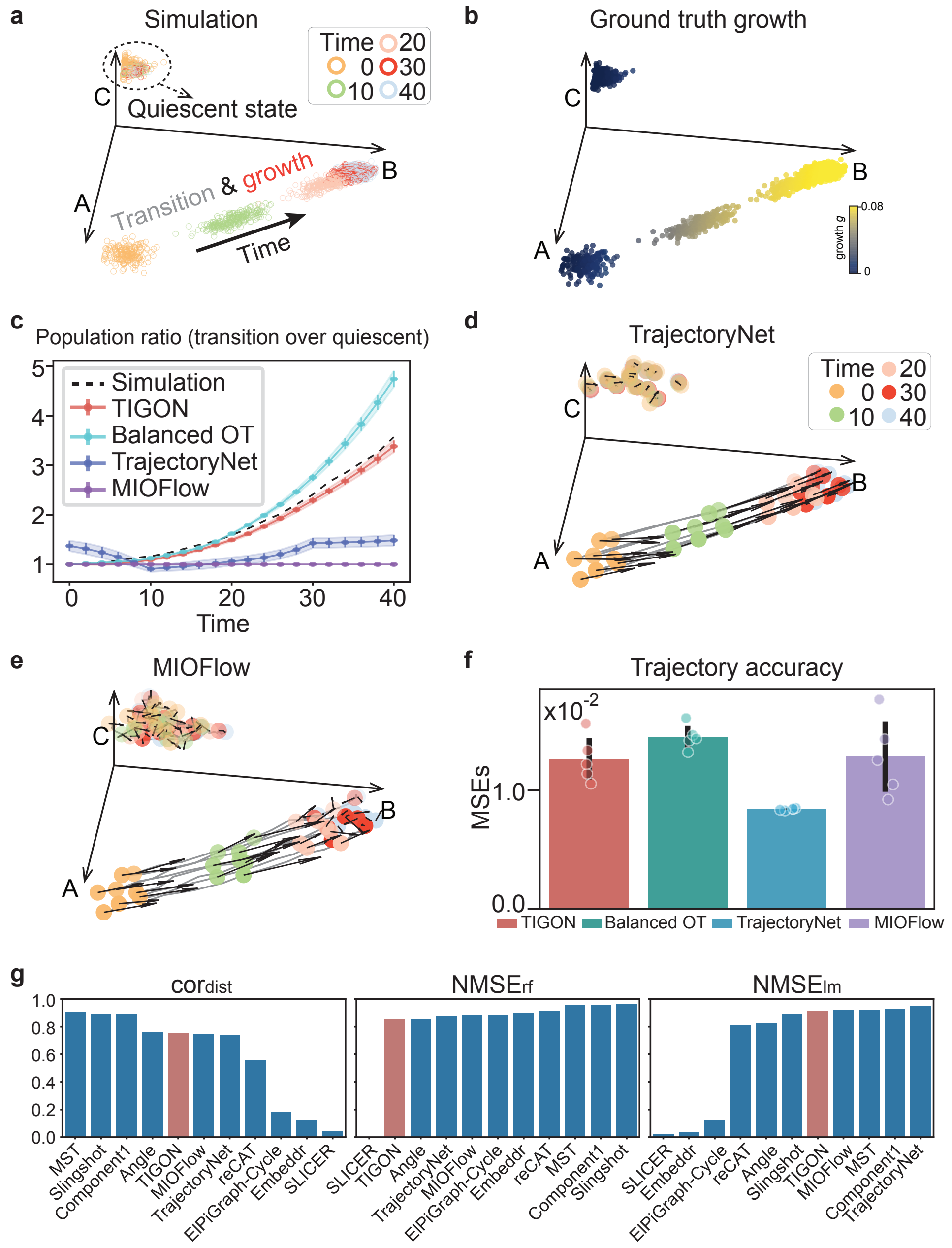


**Supplementary Figure 1: Trajectory inference benchmark on simulated data.** It is supplement to figure 2. (a) Simulation results at five time points. (b) Values of growth. Data is shown for identical cells in (a). (c) Comparisons of predicted cell population ratio between transition cells and quiescent cells as inferred by TIGON, balanced OT, TrajectoryNet, and MIOFlow. The dashed line shows the ground truth for the average cell population ratio across different time points, as obtained from the simulation. Line plots show the average cell population ratio across these time points, based on $n = 5$ independent repeats for each of the 21 time points. In the line graph, individual data points represent the average cell population ratio for the corresponding time point. Error bars represent one standard deviation from the mean, while the shaded region surrounding the line represents the range within one standard deviation above and below the mean. (d-e) Single-cell dynamics inference from (d) TrajectoryNet and (e) MIOFlow. (f) Accuracy in predicting trajectory, which is measured by mean squared errors (MSEs). The error bars show one standard deviation above and below the mean for each method from $n = 5$ independent repeats. Scatter plots show the accuracy from each repeat. (g) Comparisons over 11 trajectory inference methods. Trajectories are calculated for transition cells from simulated data, that are $\mathrm{COR}_{dist}$, $\mathrm{NMSE}_{rf}$, and $\mathrm{NMSE}_{lm}$ listed from left to right in three barplots.

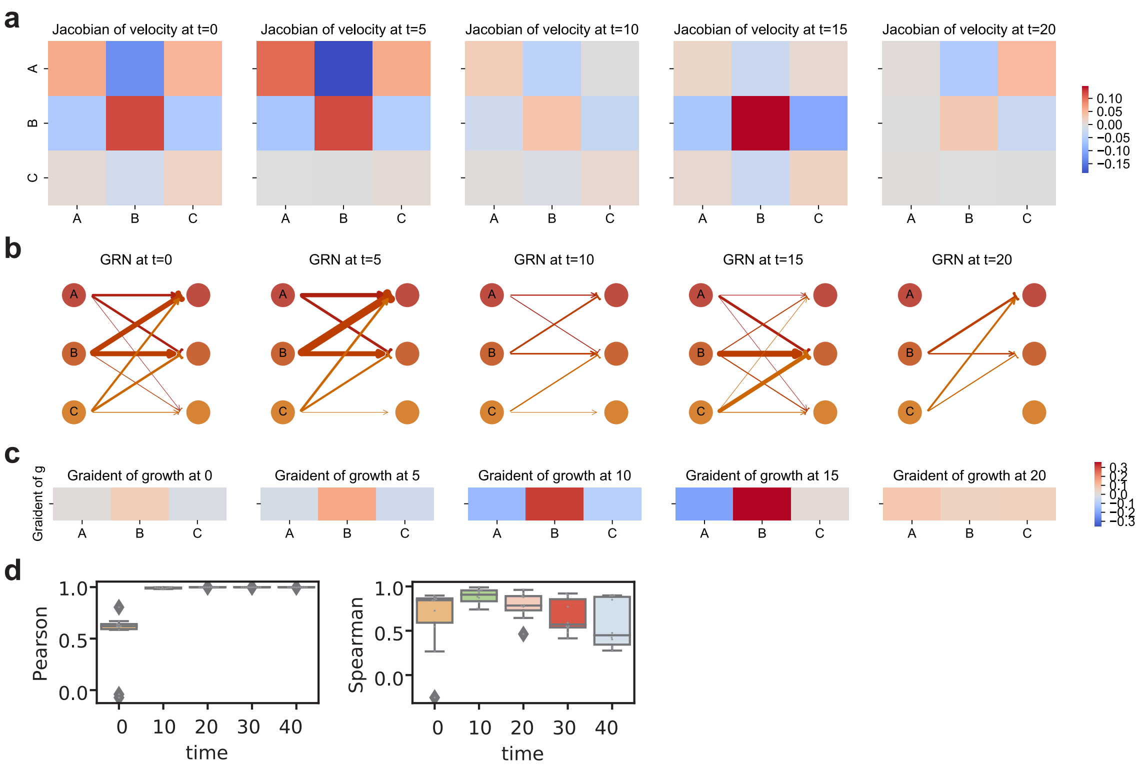


**Supplementary Figure 2: TIGON's performance on simulated data.** (a-c) Gene analysis for transition cells at different time points: (a) regulatory matrix, (b) GRN in a form of weighted directed graph and (c) gradient of growth. In (b) for GRNs, pointed arrows (blunt arrows) represent the activation (inhibition) from the source gene at starting point to the target gene at the end point, and width of lines represents the regulatory strength. (d) Predictive accuracy of growth from TIGON. The results were evaluated by Spearman and Pearson correlations based on $n = 10$ independent repeats. Sample sizes for each time point from $t = 0$ to $t = 40$ are 400, 433, 518, 682 and 914, respectively. Boxplots show the distributions of values of growth in a five-number summary where the center line shows the median, the upper and lower limits of the box show the interquartile range (IQR), spanning from the 25th to the 75th percentiles, and upper and lower whiskers show the maximum and the minimum by excluding "outliers" outside the interquartile range.

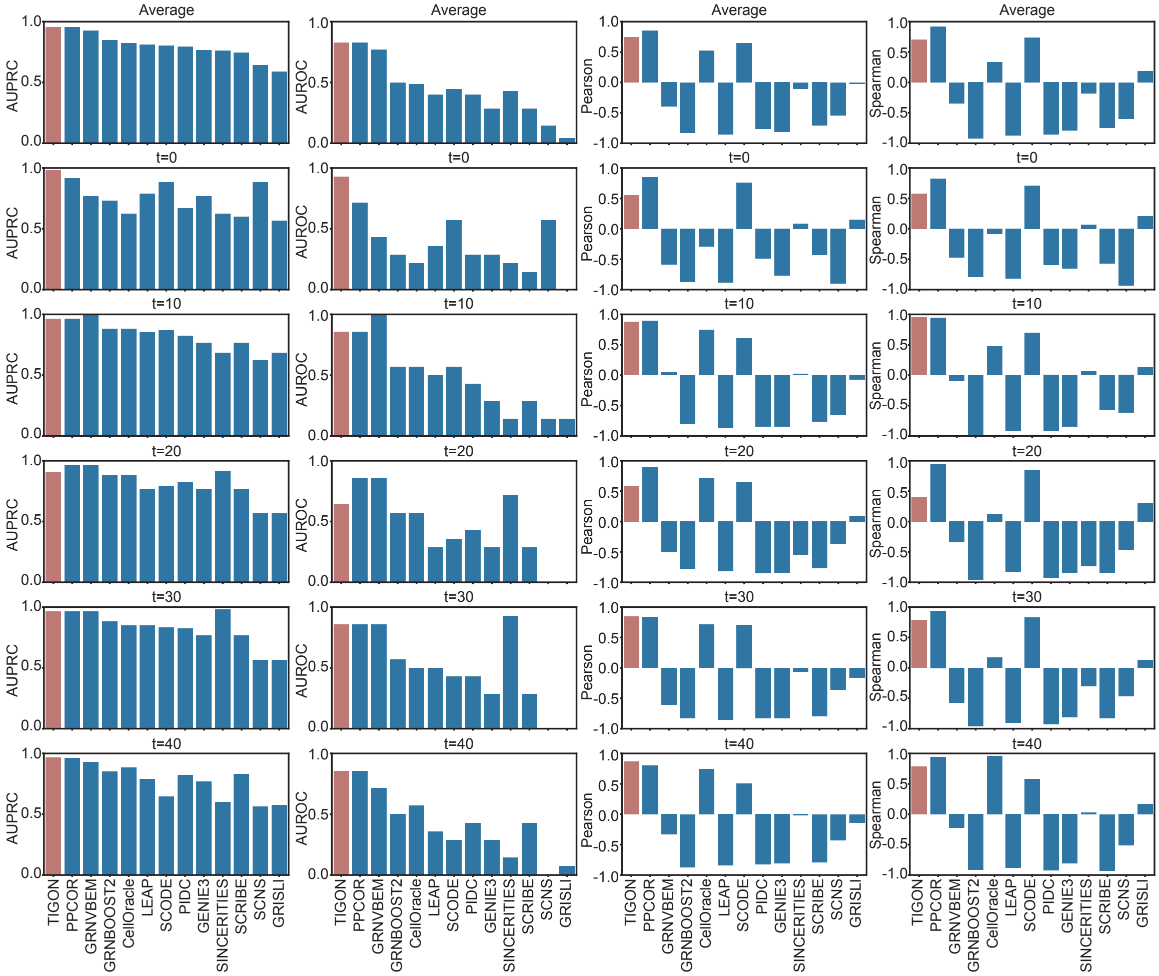


**Supplementary Figure 3: Gene regulatory network inference benchmark on simulated data.** It is supplement to figure 2j. Comparisons over 13 GRN inference methods. GRNs were inferred for transitioning cell type at five time points $t = 0,\ 10,\ 20,\ 30,\ 40$. Pearson and Spearman correlation quantify correlations between edge weights in the GRN from predictions and groundtruth. The area under precision-recall curve (AUPRC) and the area under the receiver operating characteristic (AUROC) quantify the binary classification accuracy in predicting GRN edge with directions and self-regulation but without signs, and weights. The average metrics over five time points are also shown.

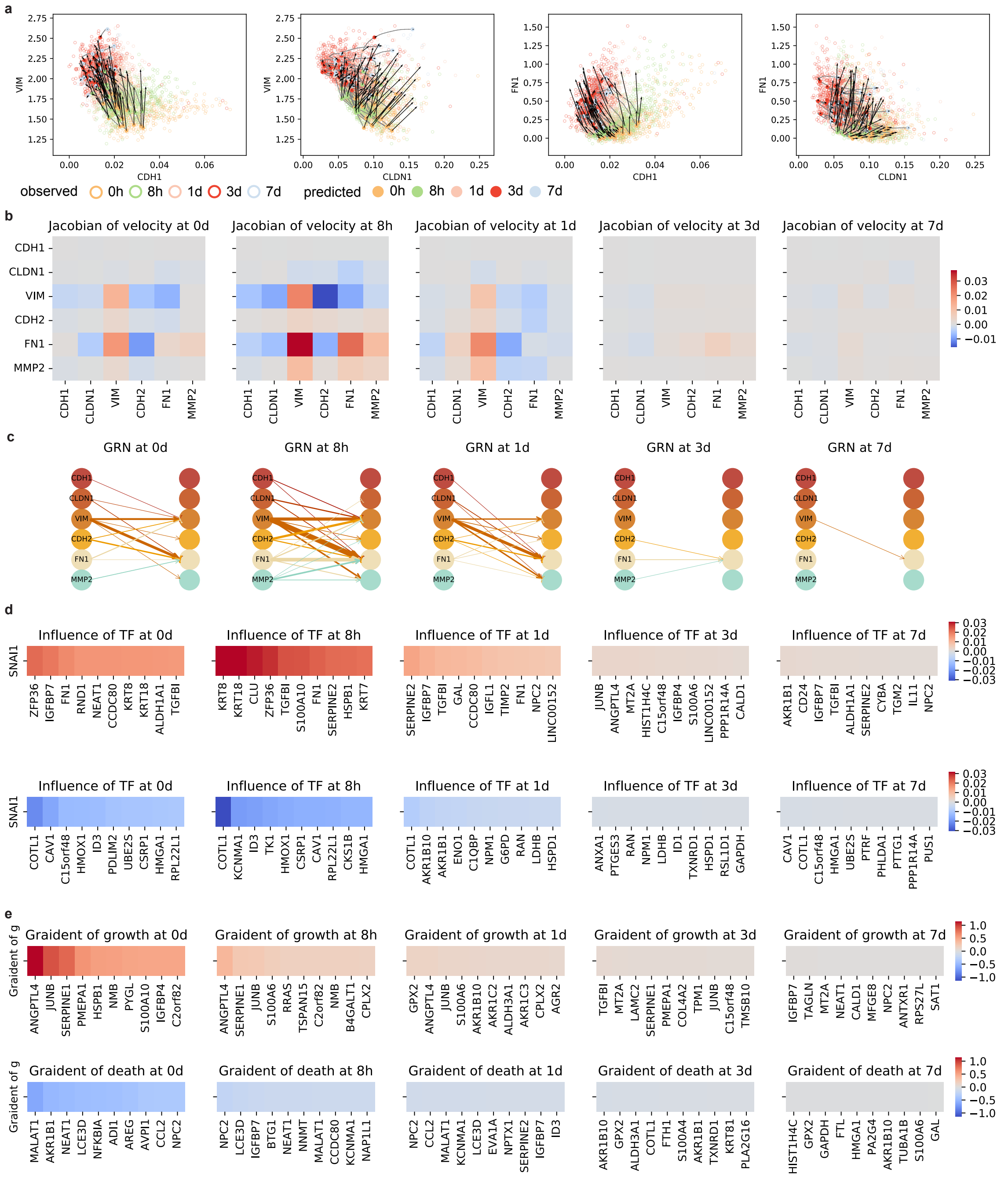


**Supplementary Figure 4: TIGON's performance on EMT dataset using ten-dimensional autoencoder latent space.** It is supplement to figure 4. (a) Trajectories and velocity for cells at gene expression space. There are 20 cells initially sampled from the density at 0 hour, where solid dots show their snapshots at 5 time points. Circles show the observed cells from the scRNA-seq data. (b) Regulatory matrix and (c) gene regulatory network (GRN) for six EMT marker genes. (d) Regulatory matrix for top 10 (top) upregulated target genes and (bottom) downregulated target genes of an EMT transcription factor (TF) SNAI1. (e) Gradient of growth for top 10 (top) growth-related genes and top 10 (bottom) death-related genes.

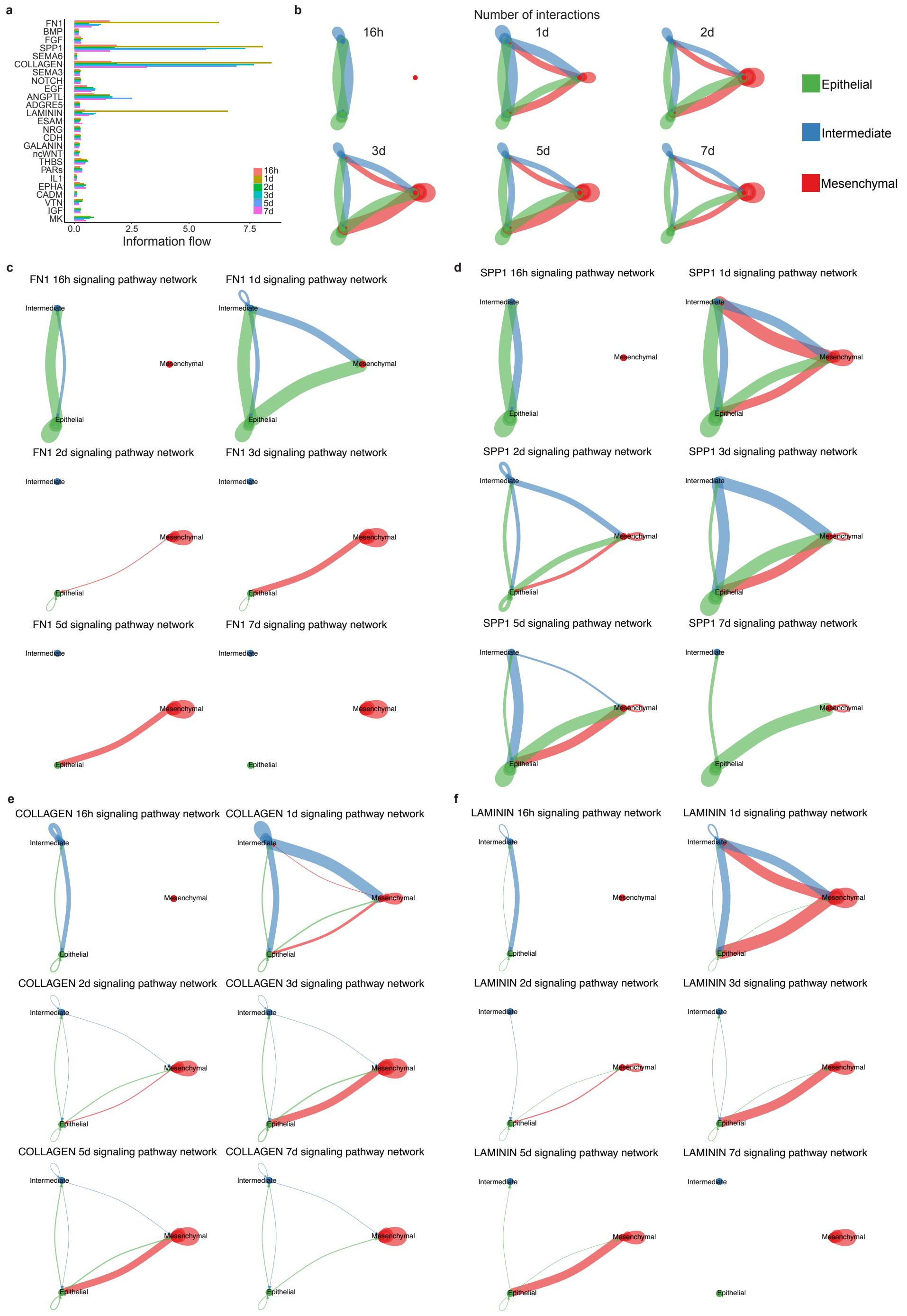


**Supplementary Figure 5: Temporal cell-cell communication from TIGON for EMT dataset.** It is supplement to figure 4h-i. (a) Barplots of information flow for all significant signaling pathways inferred by CellChat. (b) Aggregated cell-cell communication network by counting the number of links. Edge colors are consistent with the sources as sender, and edge weights are proportional to the interaction strength. Thicker edge line indicates a stronger signal. (c-f) Inferred communication networks for four signaling pathways: (c) FN1, (d) SPP1, (e) COLLAGEN, and (f) LAMININ.

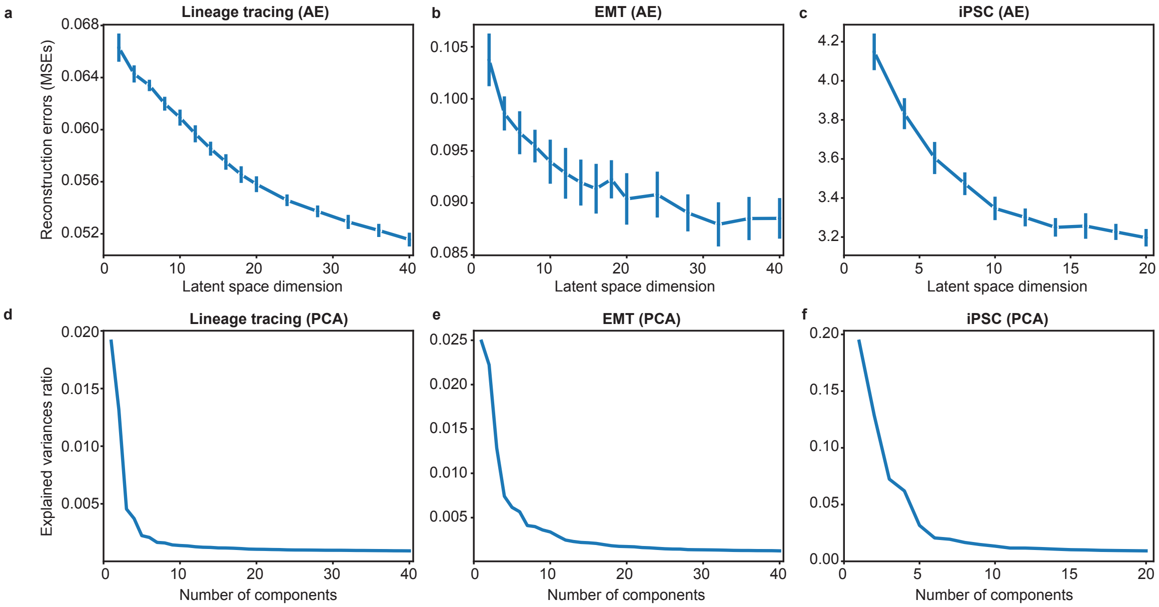


**Supplementary Figure 6: Information captured in dimension reduction methods with various dimensions.** (a-c) Reconstruction errors from autoencoder (AE) measured by mean squared errors (MSEs) versus dimension of latent space for (a) lineage tracing dataset; (b) EMT dataset; and (c) iPSCs dataset. The line plot shows MSEs derived from $n = 20$ independent repeats for each dimension of latent space. Error bars represent one standard deviation from the mean. (d-f) Explained variances ratio from principal component analysis (PCA) versus number of principal components for (d) lineage tracing dataset; (e) EMT dataset; and (f) iPSCs dataset.

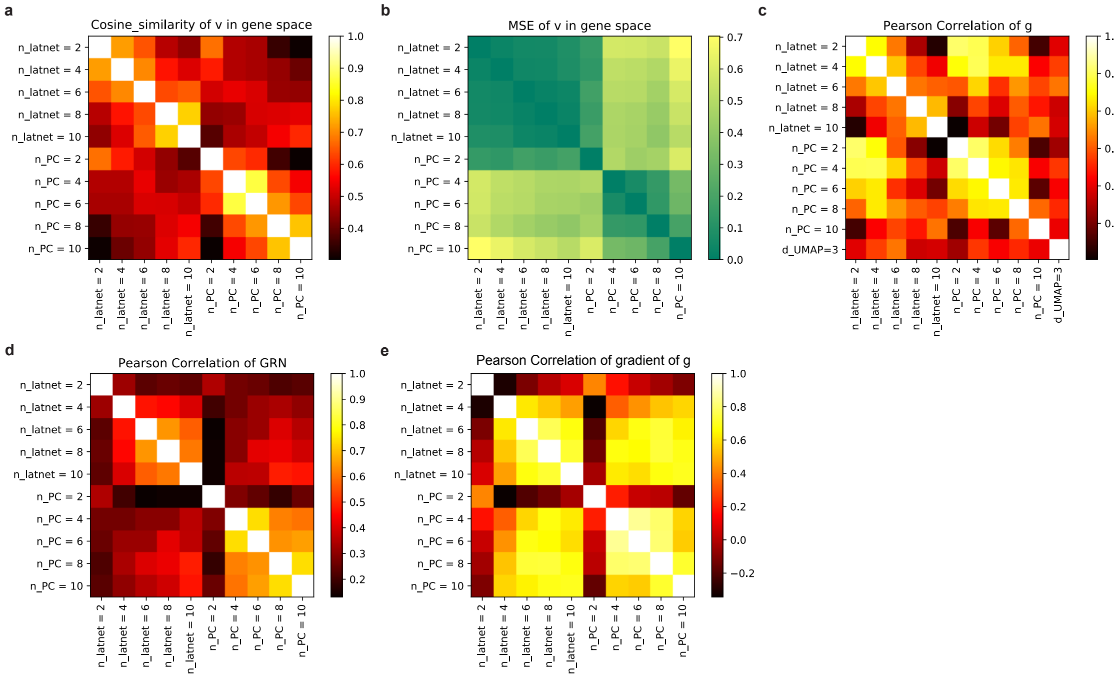


**Supplementary Figure 7: Consistency of TIGON's performance on different dimension reductions (DRs).** (a) Cosine similarity of inferred velocity for observed cells at the gene expression space for all genes. The cosine similarity was calculated for PCA and AE at different dimensions. (b) Mean squared errors (MSEs) of inferred velocity for observed cells at the gene expression space for all genes. MSEs were calculated for PCA and AE at different dimensions. (c) Pearson correlation of inferred growth of observed cells using PCA and AE at different dimensions. (d) Pearson correlation of the average gene regulatory matrices of 50 random samples at observed time points using PCA and AE at different dimensions. (e) Pearson correlation of the average gradients of growth of 50 random samples at observed time points using PCA and AE at different dimensions.

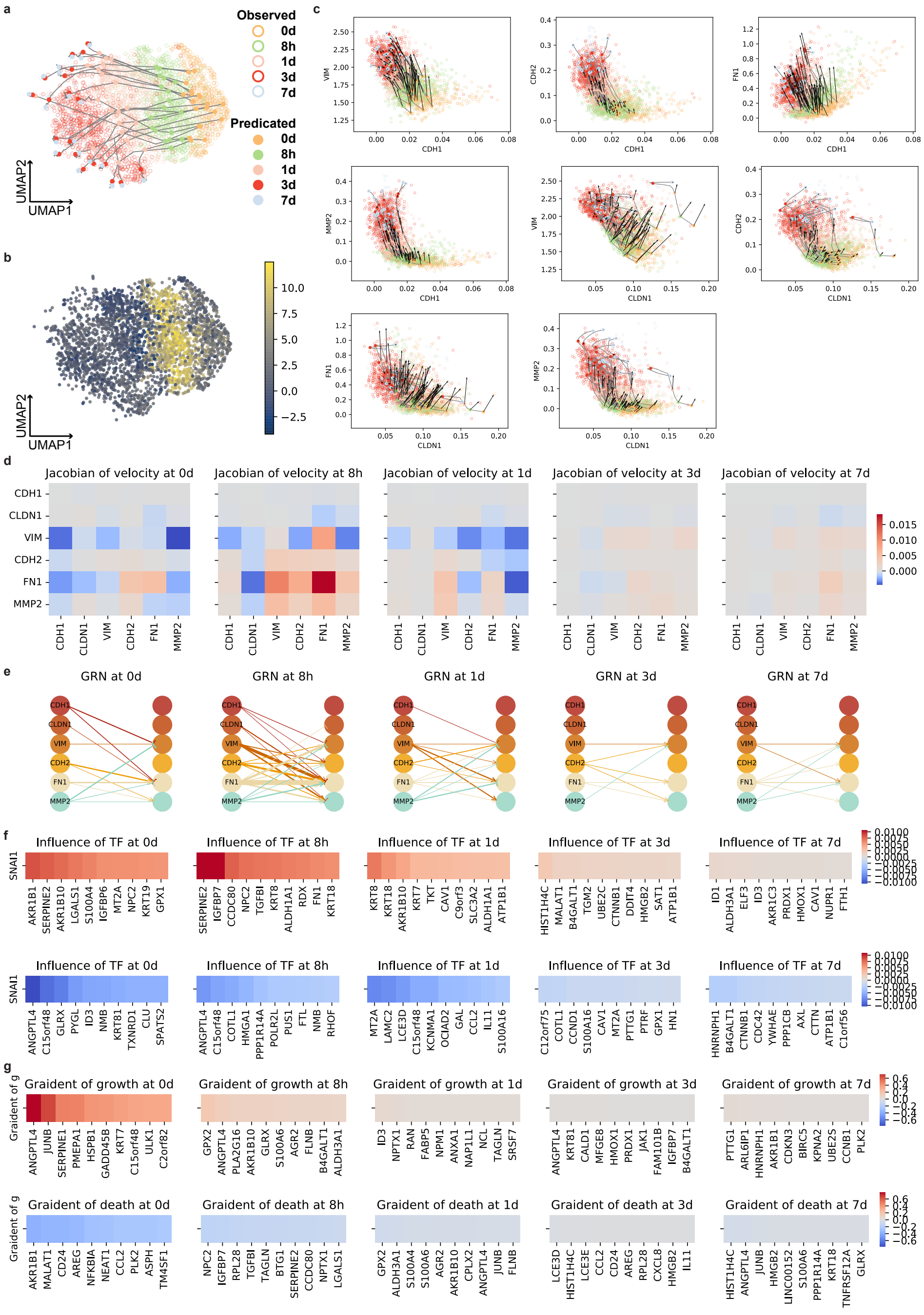

**Supplementary Figure 8: TIGON's performance on EMT dataset using eight-dimensional autoencoder latent space.** (a-b) Visualization of TIGON's outputs on UMAP space. (a) Trajectories of 20 cells that are initially sampled from the density at 0 hour, where solid dots show their snapshots at 5 time points. Circles show the observed cells from the scRNA-seq data. (b) Values of growth for all observed cells. (c) Trajectories and velocity for cells at gene expression space. Identical cells in (a) are shown in (c). (d) Regulatory matrix and (e) gene regulatory network (GRN) for six EMT marker genes. (f) Regulatory matrix for top 10 (top) upregulated target genes and (bottom) downregulated target genes of an EMT transcription factor (TF) SNAI1. (g) Gradient of growth for top 10 (top) growth-related genes and top 10 (bottom) death-related genes.

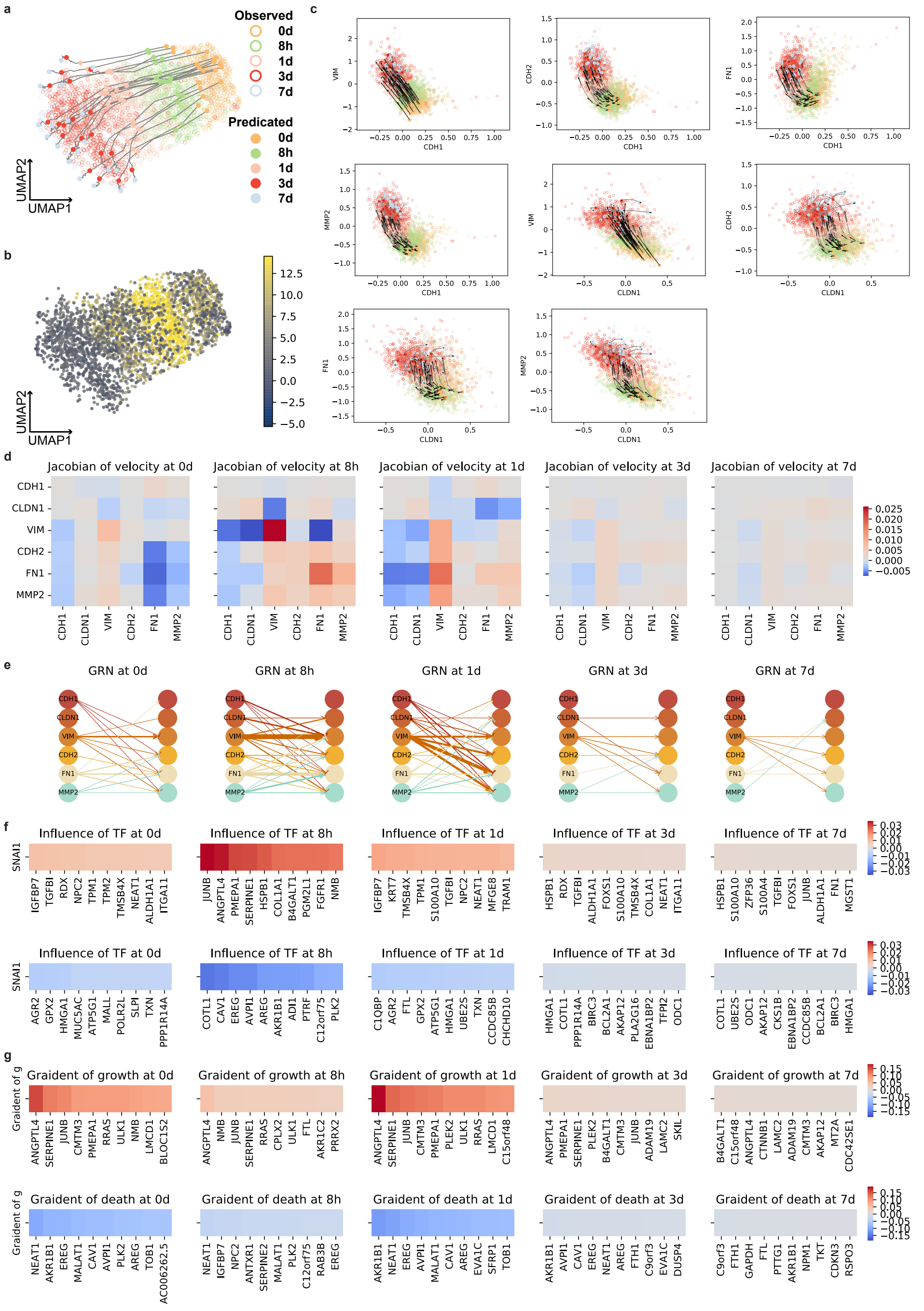


**Supplementary Figure 9: TIGON's performance on EMT dataset using first ten principal components (PCs).** (a-b) Visualization of TIGON's outputs on UMAP space. (a) Trajectories of 20 cells that are initially sampled from the density at 0 hour, where solid dots show their snapshots at 5 time points. Circles show the observed cells from the scRNA-seq data. (b) Values of growth for all observed cells. (c) Trajectories and velocity for cells at scaled gene expression space. Identical cells in (a) are shown in (c). (d) Regulatory matrix and (e) gene regulatory network (GRN) for six EMT marker genes. (f) Regulatory matrix for top 10 (top) upregulated target genes and (bottom) downregulated target genes of an EMT transcription factor (TF) SNAI1. (g) Gradient of growth for top 10 (top) growth-related genes and top 10 (bottom) death-related genes.

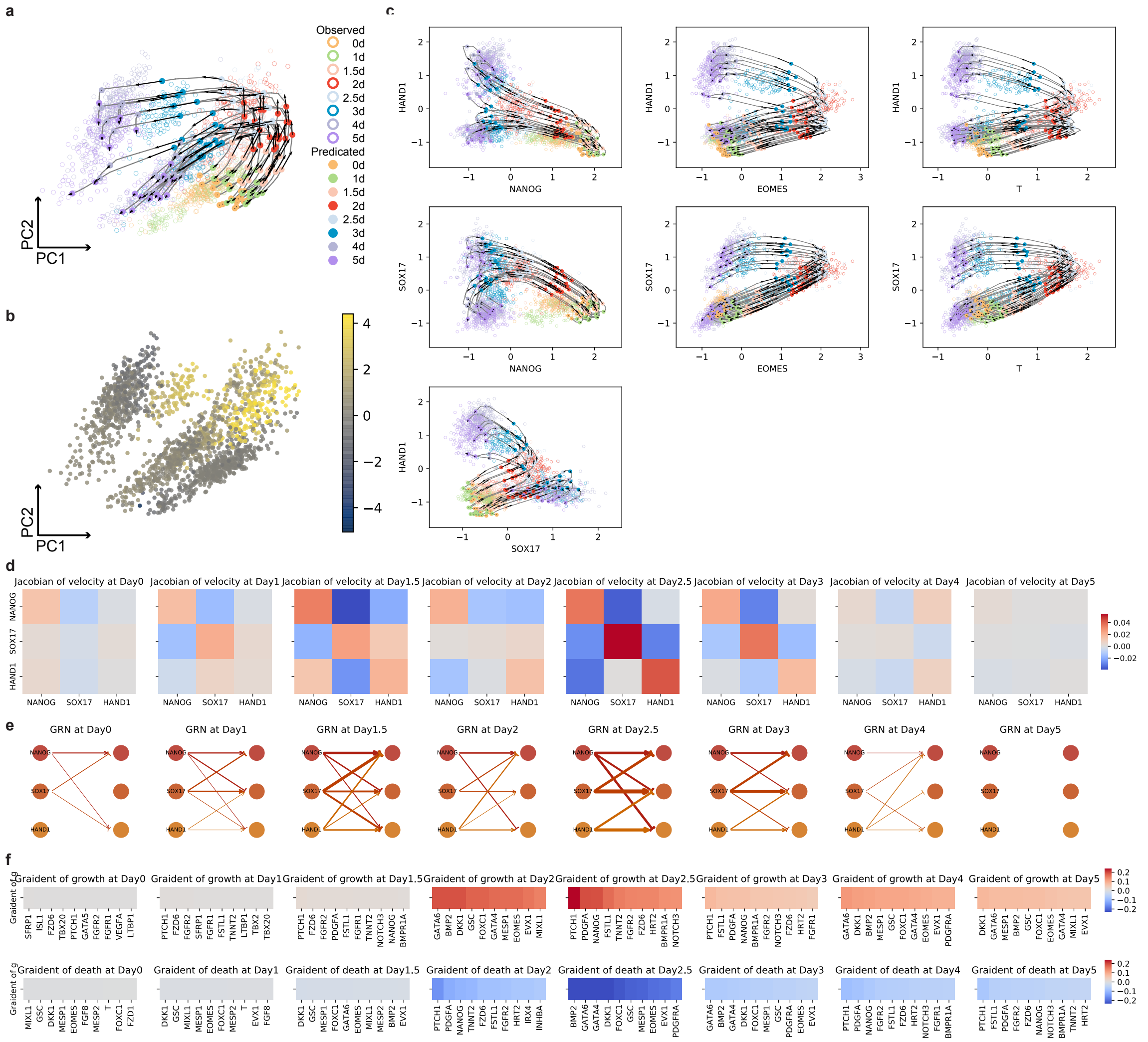

**Supplementary Figure 10: TIGON's performance on iPSCs dataset using first four principal components (PCs).** (a-b) Visualization of TIGON's outputs on first two PCs (a) Trajectories of 20 cells that are initially sampled from the density at day 0, where solid dots show their snapshots at 8 time points. Circles show the observed cells. (b) Values of growth for all observed cells. (c) Trajectories and velocity for cells at scaled gene expression space. Identical cells in (a) are shown in (c). (d) Regulatory matrix and (e) gene regulatory network (GRN) for six EMT marker genes. (f) Gradient of growth for top 10 (top) growth-related genes and top 10 (bottom) death-related genes.

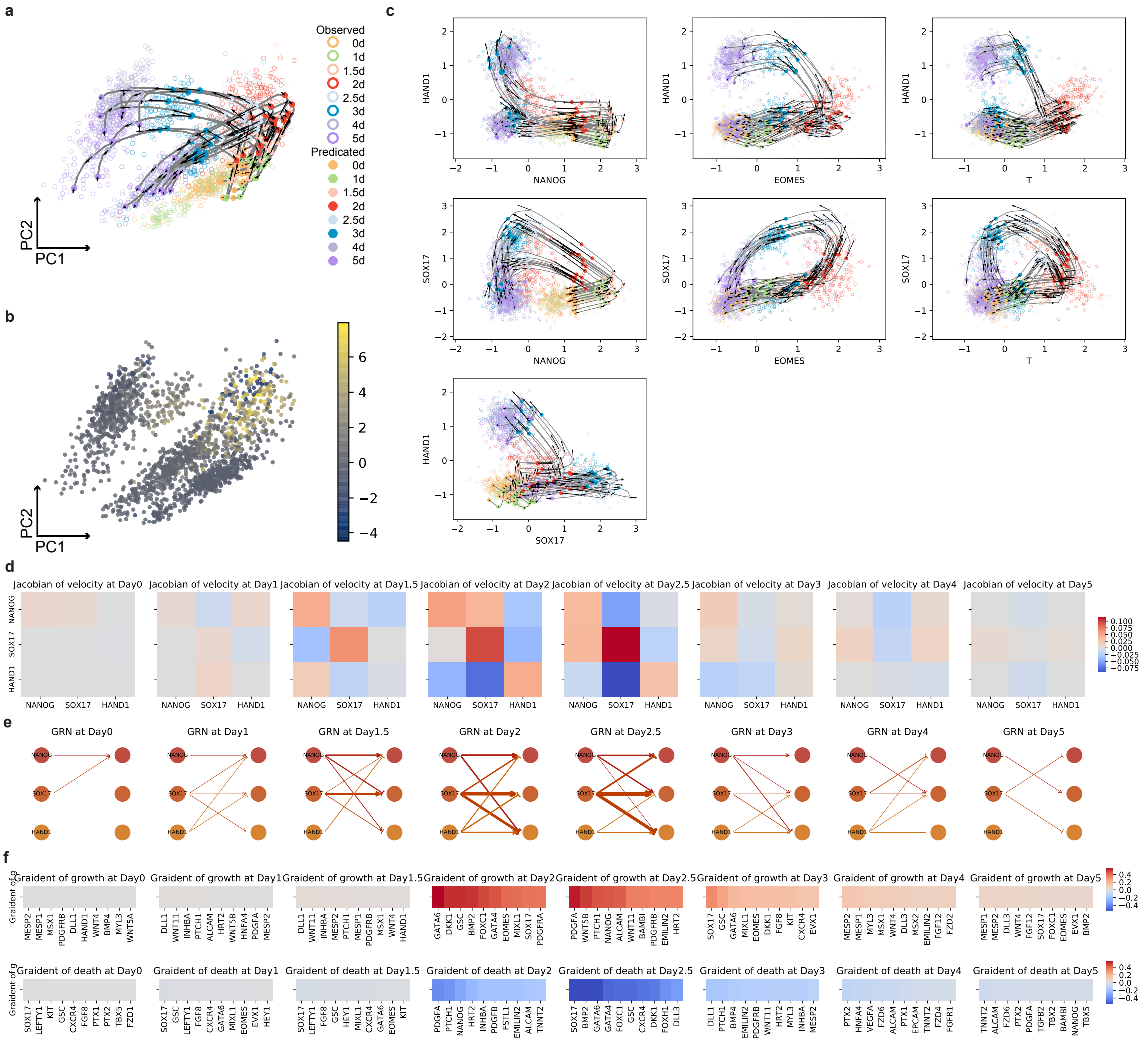


**Supplementary Figure 11: TIGON's performance on iPSCs dataset using first eight principal components (PCs).** (a-b) Visualization of TIGON's outputs on first two PCs (a) Trajectories of 20 cells that are initially sampled from the density at day 0, where solid dots show their snapshots at 8 time points. Circles show the observed cells. (b) Values of growth for all observed cells. (c) Trajectories and velocity for cells at scaled gene expression space. Identical cells in (a) are shown in (c). (d) Regulatory matrix and (e) gene regulatory network (GRN) for six EMT marker genes. (f) Gradient of growth for top 10 (top) growth-related genes and top 10 (bottom) death-related genes.

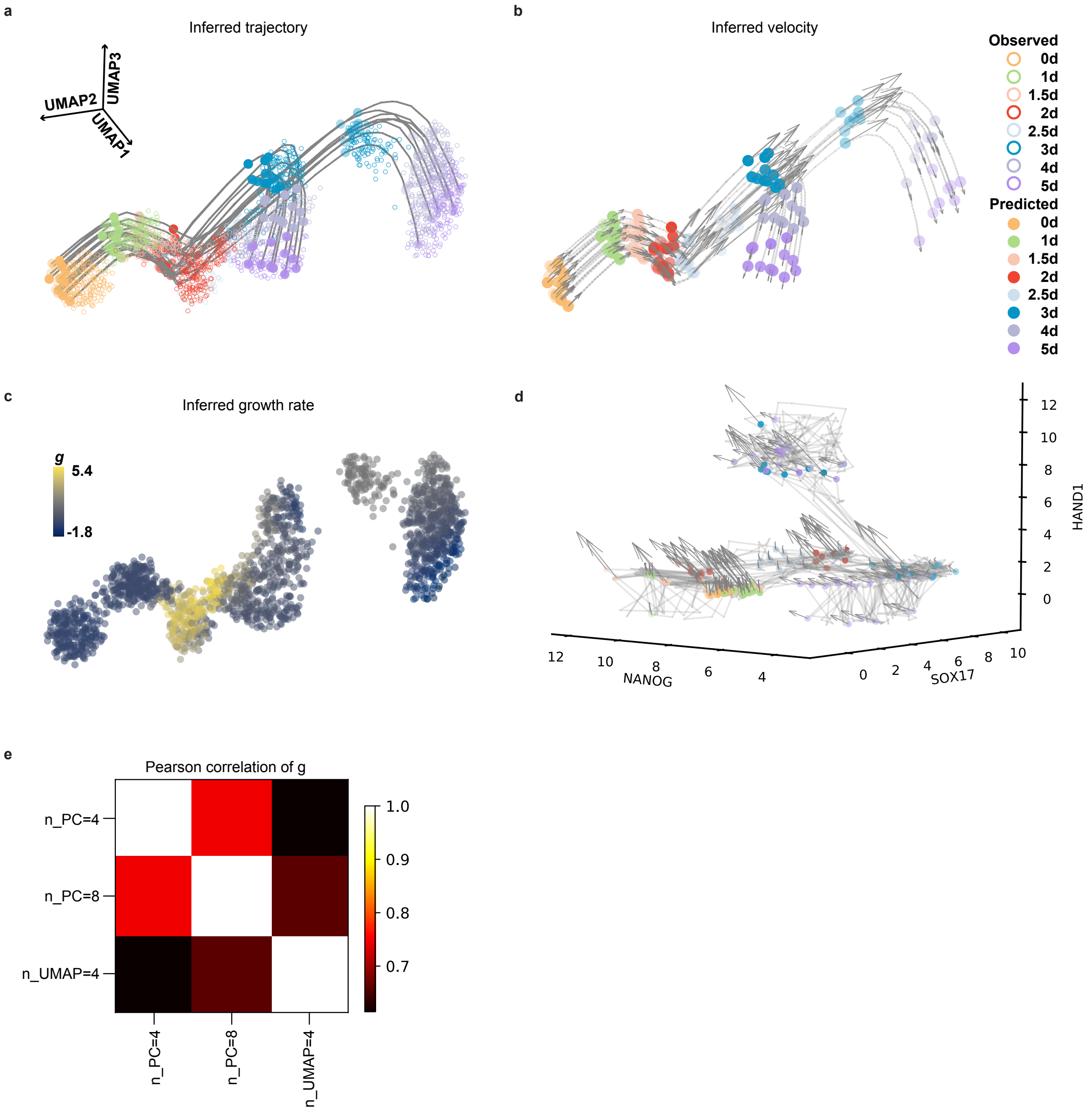


**Supplementary Figure 12: TIGON's performance on iPSCs dataset using reversible UMAP.** (a-c) Visualization of TIGON's outputs on three-dimensional UMAP space. (a-b) Velocity and trajectories of 20 cells that are initially sampled from the density at day 0, where solid dots show their snapshots at 8 time points. Circles show observed cells from the data. (c) Values of growth for 20 cells along trajectories. Identical cells were picked as in (a-b). (d) Trajectories of cells on gene expression space of three bifurcation marker genes: NANOG, HAND1, and SOX17. (e) Pearson correlation of inferred growth of observed cells from different dimension reductions.

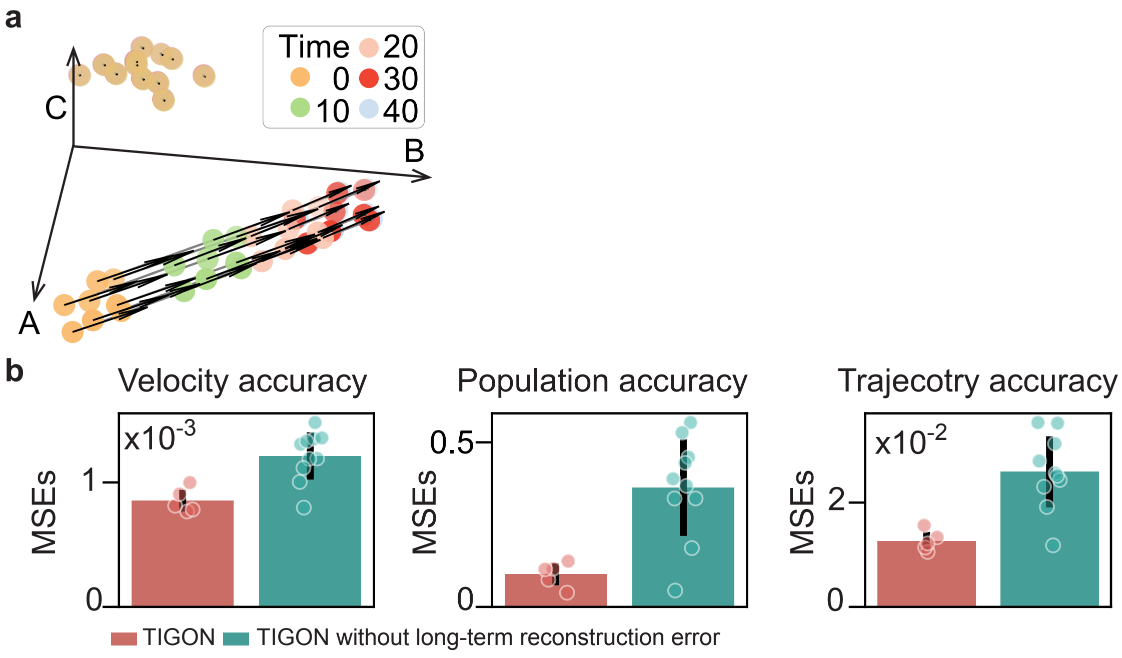


**Supplementary Figure 13: TIGON's performance without long-term reconstruction error for simulated dataset.** (a) Cellular dynamics inferred by TIGON without long-term reconstruction error for cells sampled at time=0. (b) (left) Accuracy in velocity predictions, (middle) accuracy in predicting ratio of cell population between transition cells and quiescent cells and (right) accuracy in predicting trajectory, which are measured by mean squared errors (MSEs). The error bars represent one standard deviation above and below the mean from $n = 5$ and $n = 10$ independent repeats from TIOGN and TIGON without long-term reconstruction error. Scatter plots show the accuracy from each repeat.

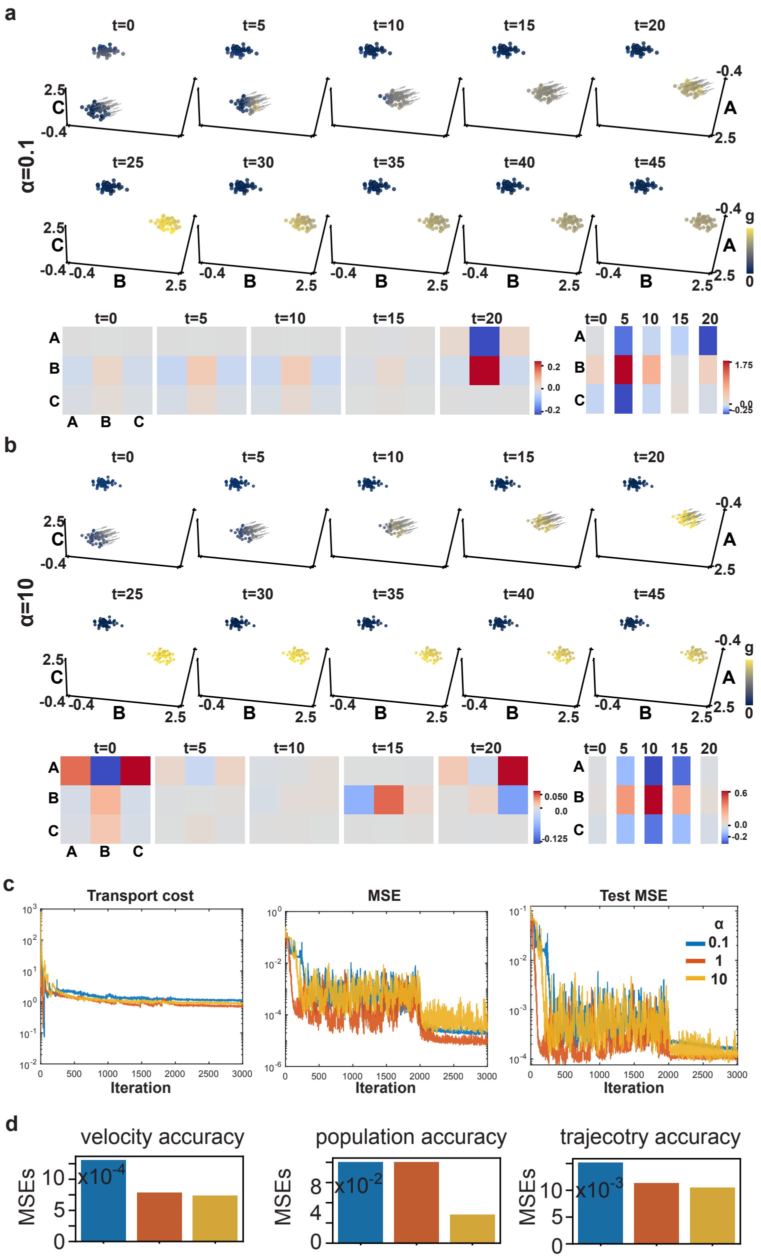

**Supplementary Figure 14: TIGON's performance when changing the weight between Wasserstein and Fisher-Rao for simulated dataset.** (a-b) (top) Cellular dynamics inferred by TIGON with weight between Wasserstein and Fisher-Rao (a) $\alpha = 0.1$ and (b) $\alpha = 10$ for 100 cells sampled at $t = 0$, (bottom left) regulatory matrix and (bottom right) gradient of growth. Each dot denotes a cell colored by estimated growth rate. Arrowed line denotes velocity with length showing the speed. (c) Transport cost, sum of mean squared errors (MSEs) between density of data and estimated density at training time points, and MSE between density of data and estimated density at middle time points $t = 5, 15, 25, 35$ along training process. (d) (left) Accuracy in velocity predictions, (middle) accuracy in predicting ratio of cell population between transition cells and quiescent cells and (right) accuracy in predicting trajectory, which are measured by MSEs.

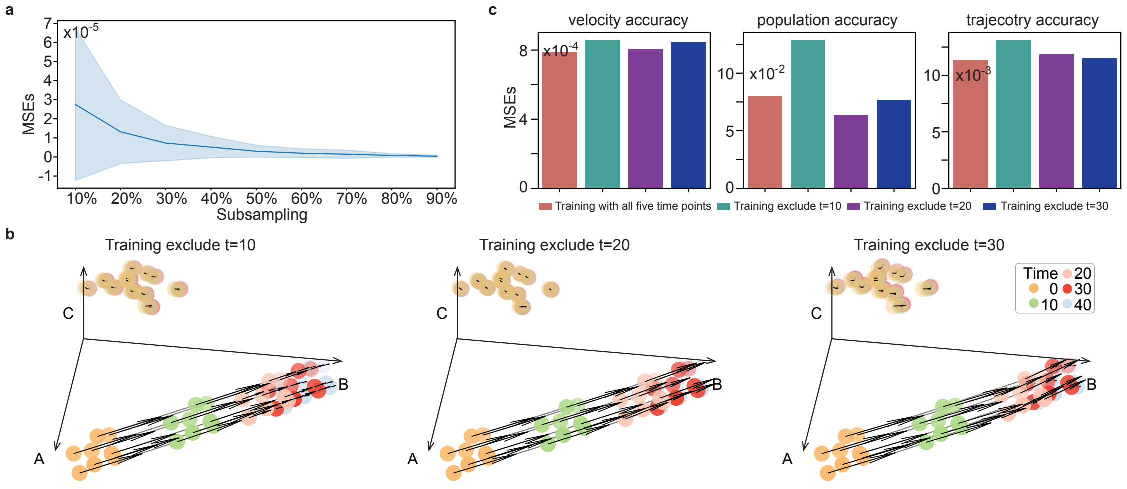


**Supplementary Figure 15: TIGON's performance using different number of samples and time points as input for simulated data.** (a) Accuracy of constructing cell densities by down-sampling samples, which is measured by mean squared errors (MSEs). The line plot shows MSEs derived from $n = 20$ independent repeats for each down-sampling. The shaded region around the line represents the range within one standard deviation above and below the mean. (b) Cellular dynamics inferred by TIGON by excluding data at one middle time point. Training data at $t = 0,\ 10,\ 20,\ 30,\ 40,\ 50$ was selected. (c) (left) Accuracy in velocity predictions, (middle) accuracy in predicting ratio of cell population between transition cells and quiescent cells and (right) accuracy in predicting trajectory, which are measured by MSEs.

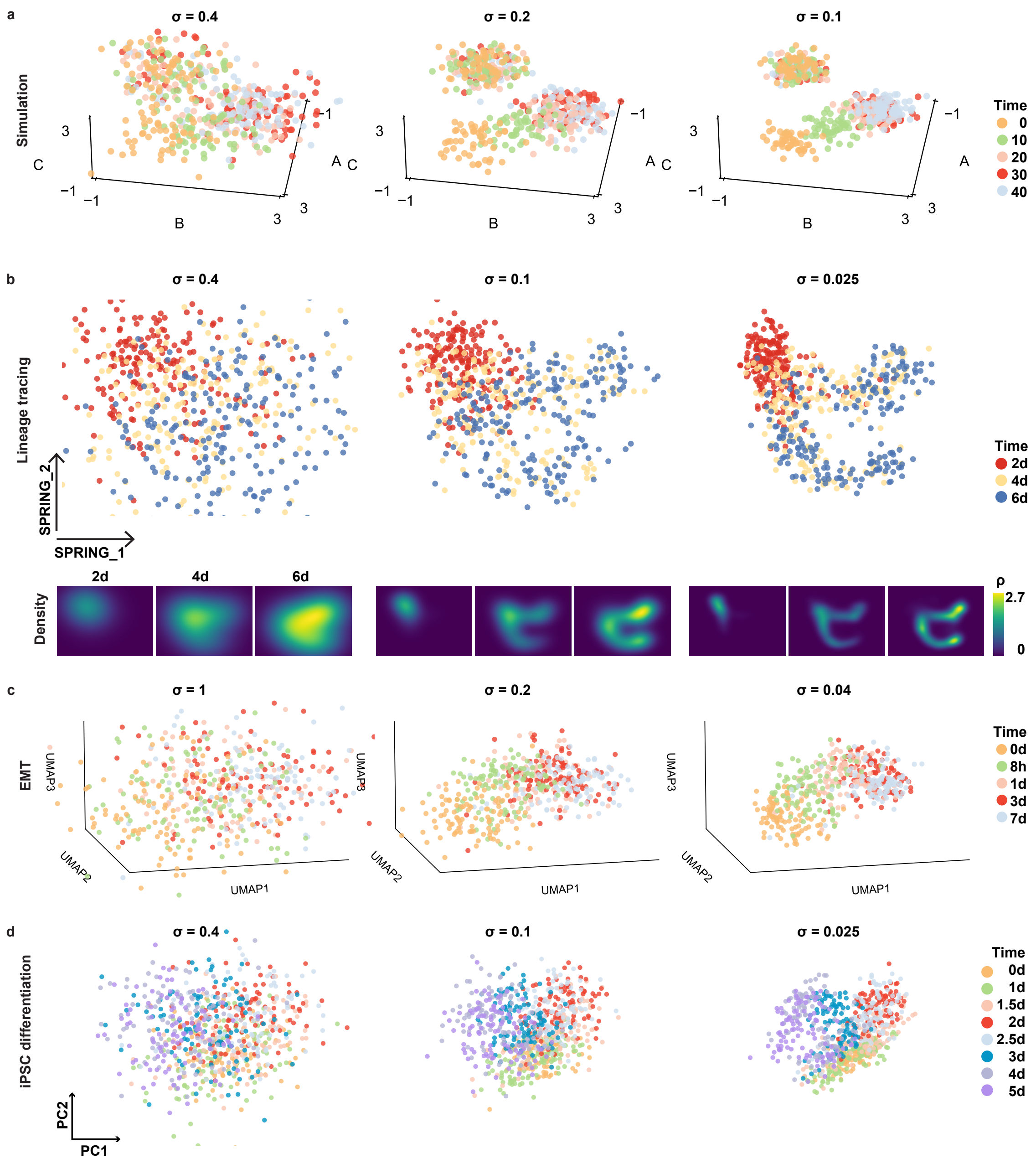


**Supplementary Figure 16: Morphology of data captured by Gaussian mixture model.** Each dot denotes a sampled cell from the Gaussian mixture model with the given standard derivation and colored by time. (a) Simulated dataset. (b) Lineage tracing dataset. (Bottom) Reconstructed densities from the Gaussian mixture model with the given standard derivation at different time points. (c) EMT dataset. (d) iPSCs differentiation dataset.

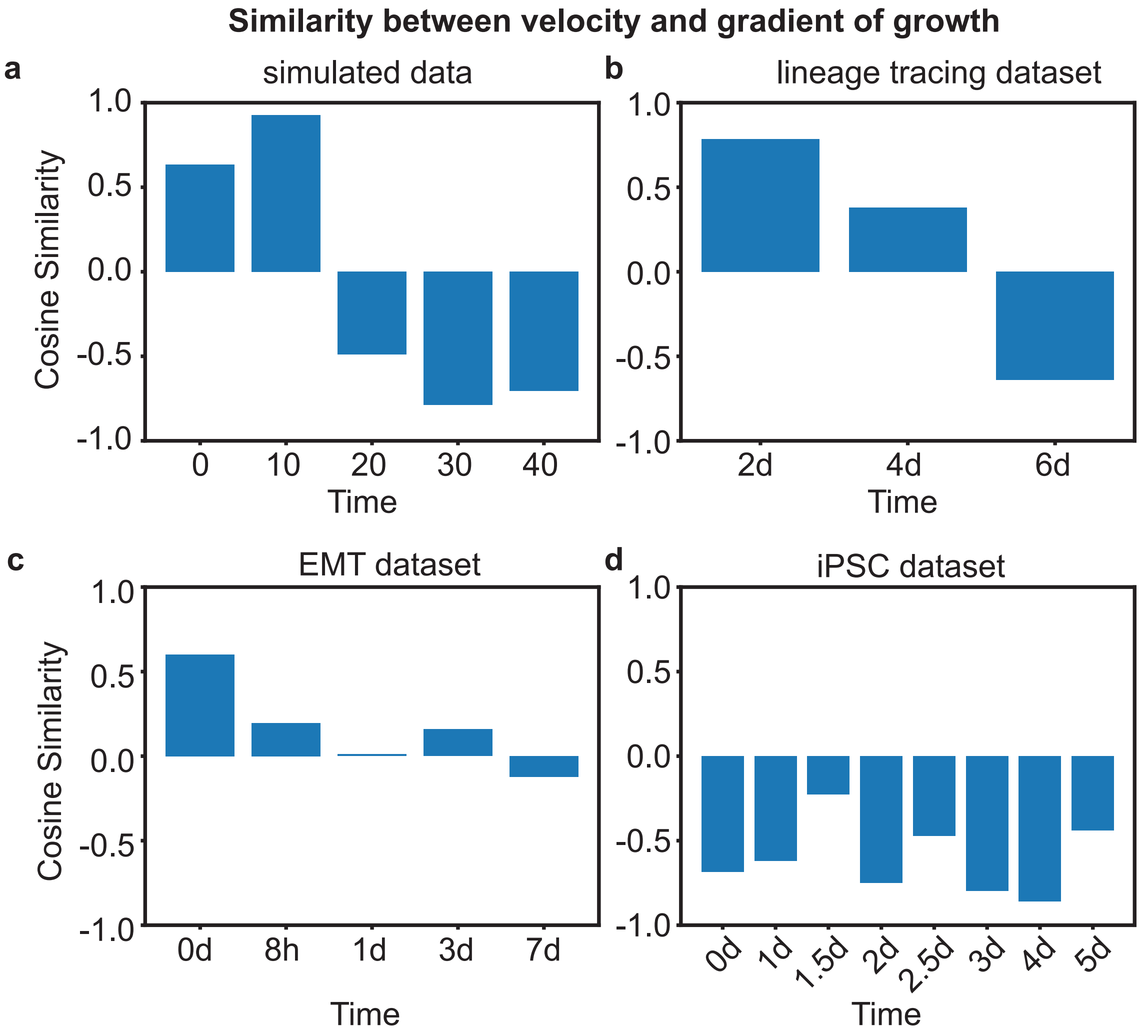


**Supplementary Figure 17: Comparisons of directionality between velocity and gradient of growth.** For each dataset, cosine similarity is calculated between velocity and gradient growth for observed cells at different time points. Datasets include (a) simulated data; (b) linage tracing dataset; (c) EMT dataset; (d) iPSCs dataset. For each dataset, calculations used TIGON results at the embedding space in main text: (a) figure 2; (b) figure 3; (c) figure 4 using UMAP embedding; and (d) figure 5.

**Algorithm** TIGON algorithm

**Require:** A series of snapshots $\left(t_1, C^1\right), \left(t_2, C^2\right), \cdots, \left(t_T, C^T\right)$ where $C^i = \left\{c_{t_i}^{(j)}\right\}_{j=1}^{N^i} \in \mathbb{R}^{N^i \times d}$. If relative cell population $\tilde{N}^i$ is not provided, $\tilde{N}^i = \frac{N^i}{N^1}$

**Ensure:** Neural networks: $(x,t) \to NN_1 \to v(x,t)$ and $(x,t) \to NN_2 \to g(x,t)$

**Preprocessing:** Using Gaussian mixture model to generate density $\rho_{t_i}$ from snapshot $C^i$

$$\rho_{t_i}(x) = \frac{\tilde{N}^i}{N^i} \sum_{j=1}^{N^i} \frac{\exp\left(-\frac{1}{2}\left(x - c_{t_i}^{(j)}\right)^T \Sigma^{-1} \left(x - c_{t_i}^{(j)}\right)\right)}{\sqrt{(2\pi)^d |\Sigma|}}, \Sigma = \sigma I \in \mathbb{R}^{d \times d}$$

**for** *epoch* from 1 to *Epochs* **do**

$Loss = 0$

**for** $i$ from $T-1$ to 1 **do**

$x_{t_{i+1}} \sim \rho_{t_{i+1}}, x_{t_{i+1}} = \left(x_{t_{i+1}}^{(1)}, x_{t_{i+1}}^{(2)}, \cdots, x_{t_{i+1}}^{(\mathrm{K})}\right)$

▷ i.i.d sampling

Integrating backward from $t_{i+1}$ to $t_i$

$$\begin{cases} \dfrac{dx}{dt} = v(x,t) \\ \dfrac{d(z(x,t))}{dt} = g(x,t) - \nabla \cdot v(x,t) \end{cases}, \begin{cases} x\left(t_{i+1}\right) = x_{t_{i+1}} \\ z\left(t_{i+1}\right) = 0 \end{cases}$$

▷ Estimate $x\left(t_i\right) = \hat{x}_{t_i}$

$z_{t_i} = \int_{t_{i+1}}^{t_i} (g(x,t) - \nabla \cdot v(x,t)) dt = \int_{t_{i+1}}^{t_i} \frac{d(\ln \rho(x,t))}{dt} dt$

▷ Intermediate variable $z_{t_i}$

$\ln \tilde{\rho}_{t_{i+1}}\left(x_{t_{i+1}}\right) = \ln \rho_{t_i}\left(\hat{x}_{t_i}\right) - z_{t_i}$

▷ Estimate $\tilde{\rho}_{t_{i+1}}$

$W_{t_i, t_{i+1}} = \left(t_{i+1} - t_i\right) \mathbb{E}_{x_i \sim \rho_{t_i}} \int_{t_i}^{t_{i+1}} \left(|v(x,t)|^2 + \alpha |g(x,t)|^2\right) e^{\int_{t_i}^t g(x,s) ds} dt$

▷ Compute transport cost

$R_{t_i, t_{i+1}} = \frac{1}{K} \sum_{j=1}^K \left[\tilde{\rho}_{t_{i+1}}\left(x_{t_{i+1}}^{(j)}\right) - \rho_{t_{i+1}}\left(x_{t_{i+1}}^{(j)}\right)\right]^2$

▷ Compute short-term reconstruction error

Integrating backward from $t_{i+1}$ to $t_1$

▷ Estimate $x\left(t_1\right) = \hat{x}_{t_1}$

$z_{t_1} = \int_{t_{i+1}}^{t_1} (g(x,t) - \nabla \cdot v(x,t)) dt = \int_{t_{i+1}}^{t_1} \frac{d(\ln \rho(x,t))}{dt} dt$

▷ Intermediate variable $z_{t_1}$

$\ln \tilde{\rho}_{t_{i+1}}\left(x_{t_{i+1}}\right) = \ln \rho_{t_1}\left(\hat{x}_{t_1}\right) - z_{t_1}$

▷ Estimate $\tilde{\rho}_{t_{i+1}}$

$R_{t_1, t_{i+1}} = \frac{1}{K} \sum_{j=1}^K \left[\tilde{\rho}_{t_{i+1}}\left(x_{t_{i+1}}^{(j)}\right) - \rho_{t_{i+1}}\left(x_{t_{i+1}}^{(j)}\right)\right]^2$

▷ Compute long-term reconstruction error

$Loss = W_{t_i, t_{i+1}} + \lambda_d R_{t_i, t_{i+1}} + \lambda_d R_{t_1, t_{i+1}}$

**end for**

Update $NN_1$ and $NN_2$ using the Adam optimizer by minimizing the *Loss*

**end for**

# Supplementary Note 1 Gene analysis utilizing reversible and differentiable dimension reduction methods

For high-dimensional data, TIGON works on a dimension reduction space to reduce computational cost. To perform gene analysis, a reversible dimension reduction method allows the reconstruction of data in low-dimensional latent space back to the high-dimensional gene expression space, furthermore, a differentiable dimension reduction method allows inference of gene regulatory network (GRN) and growth-related genes via gradient calculations. Among all methods we tested, reversible UMAP is the reversible one, and principal component analysis (PCA) and autoencoder (AE) are both reversible and differentiable. In this section, we introduce how TIGON performs gene analysis using PCA and AE.

### Reconstruction of the original data from the principal components

PCA is a dimensional reduction method that finds the orthogonal axes, i.e. principal components (PCs), that capture the maximum variance in a dataset. Let $X \in \mathbb{R}^{n\times p}$ be the dataset including $n$ samples and $p$ features. The low dimensional representation is defined by the $k$ eigenvectors with the top $p$ largest eigenvalues, $W \in \mathbb{R}^{p\times k}$. The relation between latent representation and the original space is defined as $Z = (X - \mu)W$, where $\mu$ is a vector of the mean value for each sample.

In order to reconstruct the original data from top $k$ PCs, we map the low dimensional representation $Z$ back to $p$ dimensions by multiplying $W^\intercal$ and add the mean vector $\mu$: $\widehat{X} = ZW^\intercal + \mu$. If all $p$ eigenvectors are used, then $WW^\intercal$ is the identity matrix where no dimensional reduction is performed.

We followed standard Seurat protocol [1] to normalize and scale the raw count matrix before performing PCA. So the the mean expression across cells is 0, i.e. $\mu = 0$, and the reconstructed high-dimensional space is the scaled gene expression space.

### Reconstruction of the original data from latent space of autoencoder

AE is an unsupervised deep learning method that learns a latent representation of a high-dimensional data. AE consists of an encoder $z = f(x)$ that maps input high-dimensional data $x$ to a low-dimensional representation $z$, and a decoder $\hat{x} = h(z)$ that reconstructs the high-dimensional data $x$. We took MSEs as the loss function to minimize the reconstruction loss: $\|x - \hat{x}\|^2$.

Both encoder and decoder take two multilayer perceptron (MLP) layers. Except the linear layer, each layer may also contain Batch normalization layer, non-linear ReLu activation function, and a dropout layer. Adam optimizer is used to minimize the loss function. An early stopping criterion is used. Training is stopped when the validation error on the validation set does not decrease by at least a margin of *tol* with *patience* epochs.

We used the standard Seurat protocol [1] for data preprocessing. The raw count matrix is selected via quality control, and the total count of each cell is normalized. The normalized count matrix is then log-transformed to log expression. The top 3000 highly variable genes are selected for EMT and lineage tracing data set, where $n_{input} = 3000$ is the input dimension for AE. For iPSCs dataset, the processed data was provided in a log2Ex scale with dimension $n_{input} = 96$.

Detailed architecture of AE and its hyperparameters used in this work are given in Supplementary Table 3.

## Inference of GRNs

The GRN is constructed in a directed, signed, and weighted graph with self-regulation from the regulatory matrix using the Jacobian of velocity $J = \left\{ \frac{\partial v_i}{\partial x_j} \right\}_{i,j=1}^{d}$, where $\frac{\partial v_i}{\partial x_j}$ describes the regulatory strength from source $j^{th}$ gene to target $i^{th}$ gene. This is the case when TIGON is applied to the gene expression space. If TIGON is applied to a latent space from dimension reduction, the Jacobian describes the interactive relationship between different components in the dimension reduction space. We next describe how to approximate the regulatory matrix in the gene expression space using the velocity learned in latent space.

We consider $x$ and $v$ as the state of a cell and its velocity in the original high-dimensional gene expression space. After dimension reduction, the location of the cell in the latent space is $z = f(x)$ with its velocity $\tilde{v}$ learned from the neural network $\tilde{v}(z,t) = NN_1(z,t)$ at time $t$, where $f$ denotes the function of dimension reduction such as the linear map using eigenvectors of PCA and encoder in AE. We use $\hat{x} = h(z)$ and $\hat{v} = h(z + \tilde{v}) - h(z)$ to denote the reconstructed state of a cell and its velocity in the high-dimensional gene expression space, where $h$ denotes the reverse function of dimension reduction such as decoder in AE and the linear map using transpose of eigenvectors in PCA.

The GRN requires computing $\frac{\partial v_i}{\partial x_j}$ at time $t$. By using the chain rule, we have:

$$
\begin{aligned}
\frac{\partial v_i}{\partial x_j} &\approx \frac{\partial \hat{v}_i}{\partial x_j} \\
&= \frac{\partial (h(z+\tilde{v}) - h(z))_i}{\partial x_j} \\
&= \frac{\partial (h(z+\tilde{v}) - h(z))_i}{\partial z} \frac{\partial z}{\partial x_j} \\
&= \frac{\partial (h(z+NN_1(z,t)) - h(z))_i}{\partial z} \frac{\partial f(x)}{\partial x_j} \\
&\approx \frac{\partial (h(z+NN_1(z,t)) - h(z))_i}{\partial z} \frac{\partial f(\hat{x})}{\partial \hat{x}_j} \\
&= \frac{\partial (h(z+NN_1(z,t)) - h(z))_i}{\partial z} \frac{\partial f(h(z))}{\partial h(z)_j},
\end{aligned}
\tag{1}
$$

where the two partial derivatives can be computed by the reversible and differentiable dimension reduction methods and automatic differentiation of neural networks.

## Inference of contribution of each gene to growth

The contribution of each gene to growth is assessed from the gradient of growth $\nabla g = \left\{ \frac{\partial g}{\partial x_j} \right\}_{j=1}^{d}$. To approximate $\frac{\partial g}{\partial x_j}$ from the latent space at time $t$ where the growth is learned from the neural

network $g = NN_2(z,t)$ on the latent space, we use the chain rule again:

$$\begin{aligned} \frac{\partial g}{\partial x_j} &= \frac{\partial NN_2(z,t)}{\partial z} \frac{\partial z}{x_j} \\ &= \frac{\partial NN_2(z,t)}{\partial z} \frac{\partial f(x)}{\partial x_j} \\ &\approx \frac{\partial NN_2(z,t)}{\partial z} \frac{\partial f(\hat{x})}{\partial \hat{x}_j} \\ &= \frac{\partial NN_2(z,t)}{\partial z} \frac{\partial f(h(z))}{\partial h(z)_j}, \end{aligned} \tag{2}$$

where $\frac{\partial NN_2(z,t)}{\partial z}$ can be approximated by the automatic differentiation of neural networks, and $\frac{\partial f(h(z))}{\partial h(z)_j}$ can be computed from the reversible and differentiable dimension reduction methods.

## Supplementary Note 2 Quantification and evaluating metrics

### Accuracy for predictions of cellular dynamics

The accuracy for cellular dynamics predictions is quantified by three quantities: accuracy for velocity, accuracy for trajectories and accuracy for ratio of population (figure 2 and Supplementary Figure 1). They are all quantified by mean squared errors (MSEs). For each cell with index $i$, we calculate its state $x_i(t)$, velocity $v(x_i, t)$, and density $\rho(x_i, t)$ at $K$ discrete time points: $t_j = (j-1)\Delta t,\ j = 1, \cdots, K,\ t_K = T = 40$. Accuracy for velocity is calculated as MSEs for $N = 400$ cells for $K = 21$ discrete time points between ground truth and predicted velocity:

$$\text{accuracy of velocity} = \frac{1}{NK} \sum_{i=1}^{N} \sum_{j=1}^{K} \| v^{truth}(x_i, t_j) - v^{predict}(x_i, t_j) \|^2. \tag{3}$$

Similarly, accuracy for trajectory is calculated as MSEs between ground truth and predicted trajectories:

$$\text{accuracy of trajectory} = \frac{1}{NK} \sum_{i=1}^{N} \sum_{j=1}^{K} \| x_i(t_j) - x_i^{predict}(t_j) \|^2. \tag{4}$$

We calculate the ratio of population at each time $t_j$ as the ratio between summed densities for cells at transition state over summed densities for all cells. For time $t_j$, we find index set $I$ for cells that are in transition state. Then the ratio is defined as:

$$r_j = \frac{\sum_{i \in I} \rho(x_i, t_j)}{\sum_{i=1}^{N} \rho(x_i, t_j)}. \tag{5}$$

Then the accuracy of ratio of population is calculated as MSEs between ground truth and predicted ratios:

$$\text{accuracy of cell population ratio} = \frac{1}{K} \sum_{j=1}^{K} \| r_j^{truth} - r_j^{predict} \|^2. \tag{6}$$

The ground truth of velocity, trajectory and cell population ratio is obtained from the three-gene model.

## Accuracy in gene regulatory network (GRN) inference

GRN is given as a weighted, directed, and signed graph with self-regulation. TIGON is able to infer all features of a GRN by computing the Jacobian matrix of ODEs' right hand side.

We first followed the BEELINE benchmark [2] protocol to use area under precision-recall curve (AUPRC) and the area under the receiver operating characteristic (AUROC) to quantify the GRN inference accuracy (Supplementary Figure 3). Specifically, both AUPRC and AUROC treat GRN prediction as a binary classification problem, considering directions, signs, and self-regulation within the GRN, yet without incorporating weights.

Furthermore, we also used Pearson and Spearman correlations to quantify the accuracy of GRN predictions. Specifically, these two correlations are calculated the weights of GRN edges between predictions and ground truth. This quantity treats GRN prediction as a regression problem, considering directions, signs and self-regulation within the GRN and also weights.

To calculate ground truth GRN weights, we the Jacobian matrix of the ODEs' right hand side. In the three-gene model, the right hand side for each gene is given as the following:

$$\begin{aligned} R_A &= \frac{C_A A^2 + S}{1 + C_A A^2 + H_B B^2 + H_C C^2 + S} \\ R_B &= \frac{C_B B^2 + S}{1 + H_A A^2 + C_B B^2 + H_C C^2 + S} \\ R_C &= \frac{C_C C^2}{1 + C_C C^2}. \end{aligned} \tag{7}$$

We excluded the noise term and the degradation term on the right hand side to compute the interaction relationships. The edge weight of GRN is obtained via the partial derivative. For example, the regulation of gene A on gene B is given as $J_{AB} = \frac{\partial R_B}{\partial A}$. Positive value of $J_{AB}$ indicates gene A activates B, negative value of $J_{AB}$ indicates gene A inhibits B and the absolute $|J_{AB}|$ indicates the strength of regulation. For GRN inference method, the predictive regulatory matrix $\tilde{J}$ is also calculated. For the edge without inferred weights, we set the component to be 0 in $\tilde{J}$. Then the Pearson and Spearman correlation are used to calculate the correlations between ground truth weights and inferred GRN weights (Supplementary Figure 3).

## Additional metrics for accuracy in trajectory inference

In addition to the three dynamic OT-based methods, TIGON, TrajectoryNet [3], and MIOFlow [4], we added further comparisons with several single-cell trajectory inference methods, that are inferred from pseudotime analysis. Since the pseudotime analysis provides neither trajectory along biological time nor velocity, we used three standard metrics used in a benchmark work [5]. Within the benchmark work, we chosed three metrics that do not account for bifurcation, which is the scenario presented in the simulated dataset. Specifically, they are cor_dist, NMSE_rf and NMSE_lm. cor_dist quantifies the similarity in cellular positions between predicted and ground truth trajectories. NMSE_rf and NMSE_lm quantify the accuracy of cellular positions prediction in one trajectory using positions of cells within another trajectory. We used the cells undergoing transitions in simulated data as comparisons. The velocity inferred by three dynamic OT-based methods was first translated into likely cell transitions using function scv.tl.velocity_graph in scVelo [6]. The three

dynamic OT-based methods were then considered as cell graph based methods using cell transitions as the input for dynbenchmark [5].

### Fate probability quantification

For the lineage tracing dataset [7], each cell at day 2 has a unique trajectory inferred by TIGON. To compute the probability of their descendant cells committing to Neu fate, we first calculated the fate densities for Neu and Mo cells at day 6. We used the cell fate annotation from the original work for lineage tracving data [7] in the 2-dimensional SPRING space. For Neu cells, $\{d_{6,j}^{Neu}\}_{j=1}^{N}$, and Mo cells, $\{d_{6,j}^{Mo}\}_{j=1}^{M}$, at day 6, we reconstructed fate densities for them:

$$\rho_6^{Neu}(x) = \frac{1}{N}\sum_{j=1}^{N} \frac{\exp\left(-\frac{1}{2}\left(x - d_{6,j}^{Neu}\right)^T \Sigma^{-1}\left(x - d_{6,j}^{Neu}\right)\right)}{\sqrt{(2\pi)^2|\Sigma|}} \tag{8}$$

$$\rho_6^{Mo}(x) = \frac{1}{M}\sum_{j=1}^{M} \frac{\exp\left(-\frac{1}{2}\left(x - d_{6,j}^{Mo}\right)^T \Sigma^{-1}\left(x - d_{6,j}^{Mo}\right)\right)}{\sqrt{(2\pi)^2|\Sigma|}} \tag{9}$$

Here we used diagonal covariance matrix $\Sigma = \sigma I \in \mathbb{R}^{2\times 2}$, with $\sigma = 0.025$.

For an observed cells $d_{2,j}$ at day 2, we integrated its inferred velocity $v$ forward along the trajectory to predict its state at day 6:

$$\hat{d}_{6,j} = d_{2,j} + \int_2^6 v(x(t), t)dt. \tag{10}$$

Then we utilize the predicted cell state at day 6 to calculate its Neu fate probability using the fate densities:

$$j - \text{th cell's fate probability} = \frac{\rho_6^{Neu}(\hat{d}_{6,j})}{\rho_6^{Neu}(\hat{d}_{6,j}) + \rho_6^{Mo}(\hat{d}_{6,j})}. \tag{11}$$

The clonal fate probabilities from Waddington-OT (WOT) [8], population balance analysis (PBA) [9], and FateID [10] were calculated using the predictions precomputed and made available by the original study on lineage tracing dataset [7].

### Growth inference from lineage tracing barcode or databased

For the lineage tracing data, we used the approach from [11] to estimate the value of growth $g$. The experimental data is measured at a set of time points $t_1,\ t_2, \cdots, t_T$. First, we classified cells into several groups with the identical barcode. With the same barcode, cells at earlier time points are the clonal progenitors of cells at later time points. For each group of cells with identical barcode $k$, we define a relative cell population change at $t_i$ as the ratio of cell number between $t_{i+1}$ and $t_i$, which is given as the following:

$$\tilde{n}_{t_i}^k = \frac{n_{t_{i+1}}^k}{n_{t_i}^k}. \tag{12}$$

Then all cells at $t_i$ with barcode $k$ have value of growth estimated by

$$g_{t_i}^k = \frac{\log \tilde{n}_{t_{i+1}}^k}{t_{i+1} - t_i} \tag{13}$$

Here we consider the clones from Mo and Neu trajectory and detected from day 2. Notice that the observed cells do not include all descendants since day 2 includes half of cells, day 4 includes 30% of the other half and day 6 includes the remaining cells. We adjusted the number of cells based on the sampling percentage to make sure that the number of cells are comparable among days.

For other datasets without lineage tracing barcode, we used the approach from [11] to estimate the growth rate $g$ where the birth and death scores are calculated by the mean of the z-scores of genes annotated to birth (KEGG_CELL_CYCLE or GO:0006915) and death (KEGG_APOSTOSIS or GO:0007049). The scores are then smoothed over cells after 5 iterations to obtain the birth rates $b$ and death rates $d$. The growth rate is $g = b - d$.

## Comparisons of different dimension reductions

The comparison of TIGON's performance with different dimension reductions is quantified by four quantities: consistency of velocity, growth, GRN and gradient of growth.

To compute the consistency of velocity, we projected the velocity in low-dimensional space back to the gene space, and calculated MSEs and cosine similarity between velocity inferred from different dimension reductions (DRs). Observed cells at all input time points $\{t_1, \cdots, t_T\}$ are used. In EMT dataset, the velocity at reduced space is mapped back to 3000-dimensional highly variable gene space. In iPSCs dataset, the velocity at reduced space is mapped back to the 96-dimensional gene space. These metrics for consistency between two DRs are defined as:

$$\begin{aligned}&\text{MSE of velocity in gene space between DR1 and DR2}\\ =&\frac{1}{T}\sum_{j=1}^{T}\frac{1}{N^j}\sum_{i=1}^{N^j}\|v^{DR1}(x_i,t_j) - v^{DR2}(x_i,t_j)\|^2,\end{aligned} \tag{14}$$

$$\begin{aligned}&\text{Cosine Similarity of velocity in gene space between DR1 and DR2}\\ =&\frac{1}{T}\sum_{j=1}^{T}\frac{1}{N^j}\sum_{i=1}^{N^j}\frac{\sum_{k=1}^{d} v_k^{DR1}(x_i,t_j)\cdot v_k^{DR2}(x_i,t_j)}{\sqrt{\sum_{k=1}^{d}(v_k^{DR1}(x_i,t_j))^2}\cdot\sqrt{\sum_{k=1}^{d}(v_k^{DR2}(x_i,t_j))^2}},\end{aligned} \tag{15}$$

where $N^j$ denotes the number of observed cells at time $t_j$ and $x_i$ denotes the reconstructed gene expression from low-dimensional space.

Consistency of growth inference is calculated as Pearson correlation of observed cells between two DRs. Given paired data $\{(x_1, y_1), ..., (x_n, y_n)\}$, the Pearson correlation coefficient $r_{x,y} = \frac{\sum_{i=1}^{n}(x_i-\bar{x})(y_i-\bar{y})}{\sqrt{\sum_{i=1}^{n}(x_i-\bar{x})^2\sum_{i=1}^{n}(y_i-\bar{y})^2}}$, where $\bar{x}, \bar{y}$ denotes the sample mean. The Pearson correlation for growth is then calculated as:

$$\text{Pearson correlation of growth between DR1 and DR2} = r_{g^{DR1},g^{DR2}}. \tag{16}$$

Here, we listed values of growth at different cells and time points as a vector. The paired data for Pearson correlation calculation for two DRs ia given as $\{(g^{DR1}(x_i, t_j), g^{DR2}(x_i, t_j))\}_{i=1,j=1}^{N_j,T}$.

We further introduce the metrics for quantifying the consistency of GRNs and gradient of growth inferred from different DRs. The GRN is inferred by the regulatory matrix $J(x,t) = \left\{\frac{\partial v_i(x,t)}{\partial x_j}\right\}_{i,j=1}^{d}$ on the d-dimensional gene space. To ensure the robustness, we average the regulatory matrix at all observed time points and $N = 50$ cells: $\bar{J}_{DR} = \frac{1}{NT}\sum_{j=1}^{T}\sum_{i=1}^{N} J_{DR}(x_i, t_j)$. Then the consistency of GRNs is calculated as Pearson correlation for regulatory matrices from two DRs for all components:

$$\text{Pearson correlation of GRNs between DR1 and DR2} = r_{\bar{J}_{DR1},\bar{J}_{DR2}}. \tag{17}$$

Similarly, consistency of gradient of growth is calculated as Pearson correlation for average gradient of growth $\nabla\bar{g}_{DR} = \frac{1}{NT}\sum_{j=1}^{T}\sum_{i=1}^{N}\nabla g_{DR}(x_i, t_j)$:

$$\text{Pearson correlation of gradient of growth between DR1 and DR2} = r_{\nabla\bar{g}_{DR1},\nabla\bar{g}_{DR2}}. \tag{18}$$

# Supplementary Note 3 Benchmarking TIGON against other trajectory and GRN inference methods on simulated data

This section expands comparisons between TIGON and other trajectory inference methods and GRN inference methods (figure 2g-h).

First, we compared four dynamic optimal transport (OT) based trajectory inference methods including TIGON, balanced OT, TrajectoryNet [3], and MIOFlow [4]. In this work, we refer specifically balanced OT to the framework by excluding the growth term in TIGON. In the simulated dataset, there is a group of cells making transition from state A to state B, and a group of quiescent cells staying at state C all the time (Supplementary Figure 1a). At the same time, transition cells divide with increasing rate leading to an increasing ratio of cell population between transition cells and quiescent cells (Supplementary Figure 1d). TIGON captures the behaviors in both transition and the cell population ratio (figure 2b and Supplementary Figure 1d). The other three balanced OT-based methods only capture one of the features. For balanced OT, it successfully captures the transition from state A to state B, however, it falsely predicts a transition from quiescent state C to state B (figure 2g). Since balanced OT cannot introduce growth, it utilizes the false transition to compensate the increasing ratio of population between transition and quiescent cells. On the other hand, TrajectoryNet and MIOFlow circumvent the false transition (Supplementary Figure 1d-e). As a trade-off, they infer unchanged ratio of population (Supplementary Figure 1c). Despite these three methods are able to correctly identify one behavior during the dynamics, TIGON has better accuracy on velocity and ratio of population predictions measured by MSEs (figure 2h-i). For the accuracy of trajectory, TrajectoryNet achieved the best performance and TIGON ranks the second. The different behaviors of velocity and trajectory accuracy between TIGON and TrajectoryNet are revealed by the velocity at quiescent state and initial cells at transition state. Specifically, TrajectoryNet has random velocity at quiescent state and inaccurate directions for initial cells at transition state (Supplementary Figure 1d). Those cause the inaccurate velocity predictions from TrajectoryNet. The inaccurate directions of velocity in TrajectoryNet may be caused by their assumption

that these initial cells are coming from a Gaussian distribution [4]. On the other hand, the inaccurate predictions on velocity only have minor effects on trajectory accuracy when one consider the trajectory at a long-term behavior. Indeed, TrajectoryNet shows better performance for trajectory accuracy. We further quantified the accuracy of growth predicted by TIGON. The spearman and Pearson correlations of growth are above or around 0.5 indicating the accurate growth prediction from TIGON in growth estimation (Supplementary Figure 2d).

In addition, we explored more comparisons with other trajectory inference methods with the standard metrics used in the benchmark of single-cell trajectory inference methods, i.e. pseudotime analysis [5]. We first selected 17 out of 45 methods that are ranked top three among their own wrapper type reported in the benchmark work. Next, among these 17 methods, eight of them successfully produced outputs and generated valid evaluating metrics using the cells undergoing transitions in simulated data generated from our three-gene model. These eight methods include SLICER [12], Slingshot [13], MST, Component 1, Embeddr, Angle, ElPiGraph Cycle and reCAT. In addition, three dynamic OT-based methods were included for comparisons: TIGON, TrajectoryNet [3], and MIOFlow [4]. We selected three standard metrics to assess the quality of trajectory that include cor_dist for quantifying the similarity in cellular positions between two trajectories, and NMSE_rf and NMSE_lm for quantifying the accuracy of cellular positions prediction in one trajectory using positions of cells within another trajectory. By comparing with the top methods ranked in dynbenchmark [5], three dynamic OT-based methods rank middle for cor_dist and NMSE_lm and rank top five for NMSE_rf (Supplementary Figure 1g). Furthermore, it's worth noting that the dynamic OT-based methods offer additional functionalities beyond the pseudotime-based methods. These include the application of biological time, the capability to infer velocity and single-cell trajectory, and the reconstruction of single-cell dynamics within the gene expression space.

Furthermore, we compared GRN inference in TIGON and 12 other GRN inference methods. BEELINE [2] provides a standard benchmark pipeline by implementing 13 algorithms. Applying to the simulated data, 11 of the implemented algorithms successfully generated GRNs. We also included CellOracle for comparisons [14]. Among 13 methods studied, there are eight methods allowing inference of causal GRNs (Supplementary Table 1). Six of those methods use either pseudotime or biological time, if data available, for causal inference to construct GRN. Interestingly, CellOracle provides a novel approach utilizing potential causal effects between transcription factors and target genes. TIGON also has a temporal causal inference for GRNs. Although the most of methods in comparisons infer one GRN from all cells in the data, CellOracle and TIGON are only two methods that infer cell-type specific GRNs. While CellOracle cannot infer self-regulations for a giving gene. Compared to all methods, TIGON can utilize temporal information biological time to infer cell-type specific GRNs with the capability of finding a signed, directed, and weighted GRN with self-regulation (Supplementary Table 1). We evaluated all methods on simulated data for transition cells at five time points, $t = 0,\ 10,\ 20,\ 30,\ 40$ (Supplementary Figure 3). By classifying GRN's edges with only directions, TIGON achieves the best average AUPRC and AUROC. Further comparisons for predicting GRN's edges with directions and signs, TIGON ranks 2nd and 3rd among all methods in Pearson and Spearman correlations. Although PPCOR achieves the best performance on both correlations, it always assigned the highest weights to edges for self-regulation which is coincidentally the case for the simulated model. Moreover, PPCOR cannot distinguish the directions of regulation between source and target genes.

## Supplementary Note 4 Impacts of dimension reductions on TIGON for EMT dataset

Each cell in the data usually contains thousands of genes. To efficiently use TIGON, we first perform dimension reduction, such as UMAP, PCA, and AE, to project the original data onto a low-dimensional space. In particular, PCA and AE are reversible and differentiable, allowing direct approximation of the gradient of growth and computing the regulatory matrix. To investigate how well the low-dimensional space preserves the overall information, we explored the ranking of PCs based on the percentage of variance. The 'elbow' happens earlier than 10 for the three single-cell datasets, suggesting that most of the information is captured in the first 10 PCs (Supplementary Figure 6). A similar 'elbow' plots for AE by plotting latent space dimension against reconstruction errors was also produced. The reconstruction errors saturate around 10 to 20 dimensions, suggesting that most of the information is captured when the dimension of low-dimensional space is around 10 (Supplementary Figure 6). As a conclusion, the low-dimensional space with about 10 dimensions can capture the majority high-dimensional information from the data we studied.

To study how many dimensions can provide reliable results, we studied the consistency of TIGON's performance across different dimension reduction methods using four quantities: consistency of velocity, growth, GRN and gradient of growth. This study includes PCA and AE with latent space dimensions ranging from 2 to 10 (Supplementary Figure 7-Supplementary Figure 9). In higher dimensions, the computed velocity shows consistent direction, with the value of the cosine similarity greater than or around 0.5 (Supplementary Figure 7a). As the dimensionality increases, the MSEs for velocity between two different dimensions using the same dimension reduction method diminish, suggesting better consistency (Supplementary Figure 7b). The relative large MSEs for velocity inferred between PCA and AE are due to the input gene space for PCA is the scaled gene expression space while the input for AE is normalized and log-transformed gene space without scaling, i.e. scaling mean expression of each gene across cells to be 0 and variance across cells to be 1. We then used an alternative quantitative approach by studying the correlation of inferred growth between every two different dimension reductions. For any pair, Pearson correlation is around or higher than 0.5 (Supplementary Figure 7c). Additionally, the Pearson correlation of GRNs remains positive across all dimension reduction methods, exceeding or hovering around 0.5 when the latent space dimension is greater than 2 (Supplementary Figure 7d). For growth-related genes, the correlation is similarly above 0.5 for dimensions greater than 2 (Supplementary Figure 7e). Taken together, these findings indicate that TIGON yields relatively consistent results across different dimension reductions and a wide range of latent space dimensions, and it is particularly noticeable when the dimension of the latent space exceeds 2.

In this work, we have tested TIGON on reduced space with various dimensions from 2 to 10. Higher dimension such as 20-dimensional embedding is possible, except that the computation took long time to complete. The neural networks were trained on a single Nvidia V100 (16GB) GPU. The completion time for the training process varies depending on the type of data and the dimensionality of the latent space. For simulated data, training took around 7 hours. For the single-cell datasets, the approximate training time are: 31 hours for lineage tracing data, 14 hours for EMT in a 2-dimensional latent space, 24 hours for EMT in a 10-dimensional latent space, and 72 hours for iPSCs data in either 4 or 8-dimensional latent spaces. While the training may take a long

time, it's important to note that this is a pre-training process. The pre-trained TIGON model is able to run on a personal computer for trajectory and gene analysis. To circumvent potential issues of stiff equations, we employed an adaptive step ODE solver. Using a fixed time step ODE solver could significantly trim down the training time, but the numerical stability may be a problem. Furthermore, to avoid local minima during training, we initiated the process with a large standard deviation for the Gaussian mixture model and gradually decreased it during the training phase. Optimization of the training period could also be achieved by providing suitable initial parameters, for instance, regularization constraints on velocity or growth. Such measures could notably reduce the required time for training the model.

## Supplementary Note 5 TIGON using revsersible UMAP for iPSCs dataset

In main text, we have tested TIGON using PCA for iPSCs dataset. Here we further applied TIGON to a 4-dimensional embedding space from reversible UMAP [15]. The reversible UMAP has support for inverse transform to generate a high-dimensional sample given a location in the low-dimensional embedding space. The inverse transforms allow us to project the inferred trajectories from the low-dimensional latent space back to the gene expression space. Then we compared the consistency between TIGON using PCA and reversible UMAP.

We generated the four dimensional embedding space of the data, log2Ex values of 96 genes for all cells, using the function umap.UMAP in UMAP with parameters: random_state = 15, n_components = 4, n_neighbors = 250, and min_dist = 0.83. Each axis in the four dimensional embedding space was then scaled to $[-2, 2]$ for training. The function inverse_transform was used to convert the embedding space to log2Ex gene-expression space.

From the inferred velocity and transition trajectory (Supplementary Figure 12a-b), cells at the early stage have similar direction of velocity, but the heterogeneity increases over time. At the branching time (i.e., day 3), cells show two distinct directions of velocity, and they are segregated into different cell fates. Later, cells persist the differentiation at their own fates, and two groups of cells are well-separated.

Similar to the case using 4 PCs, the largest growth rate was observed near the branching time from day 2 to day 3 (Supplementary Figure 12c). The Pearson correlations of growth learned from 4 PCs, 8 PCs and reversible UMAP are above 0.6 indicating consistency of growth inference from different dimension reductions (Supplementary Figure 12e). However, when converting the inferred trajectories back to the gene-expression space of three marker genes, the trajectories became twisted (Supplementary Figure 12d). It may be induced by some cells falling outside the convex hull of the embedding. The inverse transform function operates poorly outside the bounds of that convex hull.

## Supplementary Note 6 Necessity of including long-term reconstruction error in the loss function

TIGON offers an optimal strategy that combines short- and long-term reconstruction errors to minimize integration errors across various temporal scales. To examine the effects of long-term reconstruction error, we conducted an ablation study using simulated data, excluding the long-term reconstruction error from the training process. We then evaluated TIGON's performance in predicting velocity, trajectory, and the ratio of transitioning to quiescent cell populations.

Although the inferred trajectory and velocity appeared similar to the ground truth (Supplementary Figure 13a), the accuracy of these three metrics, as measured by MSEs, increased when the long-term reconstruction error was excluded (Supplementary Figure 13b). This surge in MSEs associated with the predictions of velocity, trajectory, and the ratio of cell population indicates the importance of incorporating long-term reconstruction errors in TIGON's training process.

## Supplementary Note 7 Exploration of weights between Wasserstein and Fisher-Rao in Wasserstein-Fisher-Rao metric

In TIGON, Wasserstein-Fisher-Rao (WFR), which simultaneously minimizes Wasserstein and Fisher-Rao metrics [16, 17], is used to constrain the cost of velocity and growth terms. The weight parameter $\alpha$ in equation (15) balances the weights between Wasserstein and Fisher-Rao metrics. In the main text, $\alpha = 1$ was examined all the time. Here we perturbed $\alpha$ values, and investigated the consistence and difference of TIGON's results on the simulated data (Supplementary Figure 14).

By perturbing $\alpha$ over two magnitudes from 0.1 to 10, we observed robust and consistent inferred dynamics for both velocity and growth. The reconstructed velocity recapitulates the cellular dynamics for two distinct cell types, that are quiescent and transition states (Supplementary Figure 14a-b) as given in the simulated data (Supplementary Figure 1a). The distinct growth patterns for two cell types are also captured by all cases, where the quiescent state maintains negligible growth rate all the time, while the transitioning state has positive growth rate. Since $\alpha$ mainly controls the weight of growth in the cost, a smaller $\alpha$ leads to relatively small growth at the later time point while results look similar in all values of $\alpha$. The inferred GRNs also show consistence for all $\alpha$ at all time examined, despite of weak regulation was observed at several time points. The mutual inhibition between gene A and B, and the self-activation for both gene A and B are captured. Furthermore, the gradient of growth consistently show gene B has strong upregulation on growth and other genes have weak correlation to growth. When $\alpha = 10$, TIGON has better accuracy in predicting ratio of cell population between transition cells and quiescent cells (Supplementary Figure 14d). TIGON has similar accuracy in predicting velocity and trajectory when $\alpha = 1$ and 10, and obtains slightly lower accuracy when $\alpha = 0.1$. Despite the additional hyperparameter $\alpha$ given in TIGON, we observed consistent results from TIGON under different $\alpha$ showing the robustness of this framework.

## Supplementary Note 8 Impacts of number of samples and time points in training data

The quality of the input data is found to be important to TIGON's performance. To examine the robustness of cell densities, i.e., input for TIGON, subject to the number of cells used in the method, we conducted a comparison between the entire population size and the a down-sampled group of cells. This study was performed on the simulated data. The comparison was based on the cell densities constructed for each group. We quantified the discrepancy in constructing cell densities on the simulated data by MSEs. The results reveal a notably low and stable MSE when the down-sampling percentage exceeded a certain threshold (i.e., 50%). This suggests that an adequate number of cells is crucial to accurately capture the cell distribution in the gene expression space (Supplementary Figure 15a).

To study the impact of the number of time points, we partitioned data at various time points into training and testing sets. The accuracy for predictions of trajectory, velocity and growth inference were examined on the simulated data at five time points (Supplementary Figure 15b-c). In each training and testing split, we picked data at one intermediate time point as testing set and the other as training set. In the three sets of training and testing splits, the inferred velocity and trajectories look similar to the ground truth, as well as that at the testing time point (Supplementary Figure 15c). The case using testing set for $t = 10$ with apparent cell transition shows slightly larger MSEs for trajectory and velocity predictions, comparing to the other two cases (Supplementary Figure 15b). The other two cases using testing set at later time point show similar magnitudes of MSEs with the case using all data as training set (Supplementary Figure 15b). This indicates that it is important to include data at critical transition stage to reconstruct accurate cellular dynamics.

In conclusion, a larger number of time points provides a denser set of snapshots capturing cellular dynamics. Sparse time points might overlook peaks in cell population, which occur within a narrow time window, leading to less accurate inferences about the dynamics. Therefore, to ensure accurate predictions of cellular dynamics, sufficient numbers of cells and time points are necessary.

## Supplementary Note 9 Synergy between gradient of growth and velocity

To examine the directionality relationship between velocity and growth gradient, we computed the cosine similarity between these two types of vectors at observed cells at different time points.

For the simulated data, the velocity and gradient of growth show consistent directions during transition (figure 2b-c). The cosine similarity of transition cells' velocity and growth gradient show positive at the early stage before $t = 20$, the time point cells are near the final state (Supplementary Figure 17a). For time points later than 20, negative cosine similarity was observed due to the same transition potential with disorganized directions for both velocity and gradient of growth.

For the lineage tracing data, we similarly observed the positive cosine similarity at the first two time points (i.e., day 2 and day 4), and a negative value at day 6 (Supplementary Figure 17b). The similar directions of two types of vectors can be clearly observed at day 2 (figure 3).

Moreover, the positive cosine similarity was observed for EMT dataset (Supplementary Figure 17c) for all time points except the final time day 7. At the most of time points, cells are undergoing

transition (figure 4). This might be the reason that EMT shows more time points with positive cosine similarity than other datasets.

From these three datasets, we clearly observed the positive cosine similarity at the transition states, suggesting a possible synergy between growth and transition processes at the early and transition stages.

However, the iPSCs dataset presents an interesting exception, as the cosine similarity remains negative throughout all the time points. This suggests possible distinct or complex dynamics in iPSCs than other datasets may involve.

| Method | Cell-type specific GRN | Causal inference | Self-regulation | Directed edge | Weighted edge | Signed edge (inhibition or activation) |
|---|---|---|---|---|---|---|
| TIGON | Yes | Biological Time | Yes | Yes | Yes | Yes |
| CellOracle [14] | Yes | ATAC-seq | No | Yes | Yes | Yes |
| GENIE3 [18] | No | No | No | Yes | Yes | No |
| GRNBoost2 [19] | No | No | No | Yes | Yes | No |
| PPCOR [20] | No | No | Yes | No | Yes | Yes |
| LEAP [21] | No | Pseudotime | No | Yes | Yes | No |
| SCODE [22] | No | Pseudotime | Yes | Yes | Yes | No |
| PIDC [23] | No | No | No | No | Yes | No |
| SINCERITIES [24] | No | Biological Time | Yes | Yes | Yes | Yes |
| SCNS [25] | No | No | Yes | Yes | No | Yes |
| GRNVBEM [26] | No | Biological Time or Pseudotime | Yes | Yes | Yes | Yes |
| SCRIBE [27] | No | Biological Time or Pseudotime | No | Yes | Yes | No |
| GRISL [28] | No | Biological Time or Pseudotime | Yes | Yes | Yes | No |

**Supplementary Table 1: Summery of GRN inference methods.** Except TIGON and CellOracle, the rest of methods were included in BeeLine benchmark [2].

| Datasets | Simulated data | Lineage tracing | EMT | iPSCs differentiation |
|---|---|---|---|---|
| Standard deviation $\sigma$ | 0.2 | 0.01 | 0.03 | 0.12 |
| Number of hidden layers in $NN_1$ | 3 | 4 | 3 | 4 |
| Dimension of hidden layers in $NN_1$ | 16 | 16 | 16 | 16 |
| Number of hidden layers in $NN_2$ | 2 | 2 | 2 | 2 |
| Dimension of hidden layers in $NN_2$ | 16 | 16 | 16 | 16 |
| Learning rate | 0.05 | 0.007 | 0.003 | 0.003 |
| Sample size | 1000 | 500 | 100 | 1000 |
| Number of Epochs | 3000 | 8000 | 5000 | 20000 |

**Supplementary Table 2: List of hyperparameters used in the training process.** TIGON uses two neural networks approximate velocity $v(x,t) \approx NN_1(x,t)$ and growth rate $g(x,t) \approx NN_2(x,t)$, respectively.

| Layer | Layer composition |
|---|---|
| Encoder layer 1 | Linear($n_{input}$, $n_{hidden}$), BatchNorm1d, ReLU, Dropout (0.2) |
| Encoder layer 2 | Linear($n_{hidden}$,$n_{latent}$), BatchNorm1d, Dropout (0.2) |
| Decoder layer 1 | Linear($n_{latent}$, $n_{hidden}$), BatchNorm1d, ReLU, Dropout (0.2) |
| Decoder layer 2 | Linear($n_{hidden}$,$n_{input}$), BatchNorm1d, Dropout (0.2) |
| **Trainer parameter** | **Values** |
| Optimizer | Adam |
| Batch size | 32 |
| maximum epoch | 500 |
| weight decay | $10^{-4}$ |
| validation ratio | 0.1 |
| *patience* | 30 |
| *tol* | $10^{-2}$ |
| **Dataset** | **Specific parameters** |
| lineage tracing | $n_{input} = 3000$, $n_{hidden} = 300$, Learning rate: $10^{-3}$ |
| EMT | $n_{input} = 3000$, $n_{hidden} = 300$, Learning rate: $10^{-3}$ |
| iPSCs | $n_{input} = 96$, $n_{hidden} = 48$, Learning rate: $10^{-2}$ |

**Supplementary Table 3: Autoencoder (AE) architecture and hyperparameters.** Layer composition describes the layer component in a sequential order.

# References


[1] Tim Stuart, Andrew Butler, Paul Hoffman, Christoph Hafemeister, Efthymia Papalexi, William M Mauck, Yuhan Hao, Marlon Stoeckius, Peter Smibert, and Rahul Satija. Comprehensive integration of single-cell data. *Cell*, 177(7):1888–1902, 2019.

[2] Aditya Pratapa, Amogh P Jalihal, Jeffrey N Law, Aditya Bharadwaj, and TM Murali. Benchmarking algorithms for gene regulatory network inference from single-cell transcriptomic data. *Nature methods*, 17(2):147–154, 2020.

[3] Alexander Tong, Jessie Huang, Guy Wolf, David Van Dijk, and Smita Krishnaswamy. Trajectorynet: A dynamic optimal transport network for modeling cellular dynamics. In *International conference on machine learning*, pages 9526–9536. PMLR, 2020.

[4] Guillaume Huguet, Daniel Sumner Magruder, Oluwadamilola Fasina, Alexander Tong, Manik Kuchroo, Guy Wolf, and Smita Krishnaswamy. Manifold interpolating optimal-transport flows for trajectory inference. *arXiv preprint arXiv:2206.14928*, 2022.

[5] Wouter Saelens, Robrecht Cannoodt, Helena Todorov, and Yvan Saeys. A comparison of single-cell trajectory inference methods. *Nature biotechnology*, 37(5):547–554, 2019.

[6] Volker Bergen, Marius Lange, Stefan Peidli, F Alexander Wolf, and Fabian J Theis. Generalizing rna velocity to transient cell states through dynamical modeling. *Nature biotechnology*, 38(12):1408–1414, 2020.

[7] Caleb Weinreb, Alejo Rodriguez-Fraticelli, Fernando D Camargo, and Allon M Klein. Lineage tracing on transcriptional landscapes links state to fate during differentiation. *Science*, 367 (6479):eaaw3381, 2020.

[8] Geoffrey Schiebinger, Jian Shu, Marcin Tabaka, Brian Cleary, Vidya Subramanian, Aryeh Solomon, Joshua Gould, Siyan Liu, Stacie Lin, Peter Berube, et al. Optimal-transport analysis of single-cell gene expression identifies developmental trajectories in reprogramming. *Cell*, 176 (4):928–943, 2019.

[9] Caleb Weinreb, Samuel Wolock, Betsabeh K Tusi, Merav Socolovsky, and Allon M Klein. Fundamental limits on dynamic inference from single-cell snapshots. *Proceedings of the National Academy of Sciences*, 115(10):E2467–E2476, 2018.

[10] Josip S Herman, Dominic Grün, et al. Fateid infers cell fate bias in multipotent progenitors from single-cell rna-seq data. *Nature methods*, 15(5):379–386, 2018.

[11] Grace Hui Ting Yeo, Sachit D Saksena, and David K Gifford. Generative modeling of single-cell time series with prescient enables prediction of cell trajectories with interventions. *Nature communications*, 12(1):1–12, 2021.

[12] Joshua D Welch, Alexander J Hartemink, and Jan F Prins. Slicer: inferring branched, nonlinear cellular trajectories from single cell rna-seq data. *Genome biology*, 17(1):1–15, 2016.

[13] Kelly Street, Davide Risso, Russell B Fletcher, Diya Das, John Ngai, Nir Yosef, Elizabeth Purdom, and Sandrine Dudoit. Slingshot: cell lineage and pseudotime inference for single-cell transcriptomics. *BMC genomics*, 19:1–16, 2018.

[14] Kenji Kamimoto, Blerta Stringa, Christy M Hoffmann, Kunal Jindal, Lilianna Solnica-Krezel, and Samantha A Morris. Dissecting cell identity via network inference and in silico gene perturbation. *Nature*, 614(7949):742–751, 2023.

[15] Leland McInnes, John Healy, Nathaniel Saul, and Lukas Grossberger. Umap: Uniform manifold approximation and projection. *The Journal of Open Source Software*, 3(29):861, 2018.

[16] Lenaic Chizat, Gabriel Peyré, Bernhard Schmitzer, and François-Xavier Vialard. An interpolating distance between optimal transport and fisher–rao metrics. *Foundations of Computational Mathematics*, 18(1):1–44, 2018.

[17] Lenaic Chizat, Gabriel Peyré, Bernhard Schmitzer, and François-Xavier Vialard. Unbalanced optimal transport: Dynamic and kantorovich formulations. *Journal of Functional Analysis*, 274(11):3090–3123, 2018.

[18] Vân Anh Huynh-Thu, Alexandre Irrthum, Louis Wehenkel, and Pierre Geurts. Inferring regulatory networks from expression data using tree-based methods. *PloS one*, 5(9):e12776, 2010.

[19] Thomas Moerman, Sara Aibar Santos, Carmen Bravo González-Blas, Jaak Simm, Yves Moreau, Jan Aerts, and Stein Aerts. Grnboost2 and arboreto: efficient and scalable inference of gene regulatory networks. *Bioinformatics*, 35(12):2159–2161, 2019.

[20] Seongho Kim. ppcor: an r package for a fast calculation to semi-partial correlation coefficients. *Communications for statistical applications and methods*, 22(6):665, 2015.

[21] Alicia T Specht and Jun Li. Leap: constructing gene co-expression networks for single-cell rna-sequencing data using pseudotime ordering. *Bioinformatics*, 33(5):764–766, 2017.

[22] Hirotaka Matsumoto, Hisanori Kiryu, Chikara Furusawa, Minoru SH Ko, Shigeru BH Ko, Norio Gouda, Tetsutaro Hayashi, and Itoshi Nikaido. Scode: an efficient regulatory network inference algorithm from single-cell rna-seq during differentiation. *Bioinformatics*, 33(15): 2314–2321, 2017.

[23] Thalia E Chan, Michael PH Stumpf, and Ann C Babtie. Gene regulatory network inference from single-cell data using multivariate information measures. *Cell systems*, 5(3):251–267, 2017.

[24] Nan Papili Gao, SM Minhaz Ud-Dean, Olivier Gandrillon, and Rudiyanto Gunawan. Sinceri-ties: inferring gene regulatory networks from time-stamped single cell transcriptional expression profiles. *Bioinformatics*, 34(2):258–266, 2018.

[25] Steven Woodhouse, Nir Piterman, Christoph M Wintersteiger, Berthold Göttgens, and Jasmin Fisher. Scns: a graphical tool for reconstructing executable regulatory networks from single-cell genomic data. *BMC systems biology*, 12:1–7, 2018.

[26] Manuel Sanchez-Castillo, David Blanco, Isabel M Tienda-Luna, MC Carrion, and Yufei Huang. A bayesian framework for the inference of gene regulatory networks from time and pseudo-time series data. *Bioinformatics*, 34(6):964–970, 2018.

[27] Xiaojie Qiu, Arman Rahimzamani, Li Wang, Bingcheng Ren, Qi Mao, Timothy Durham, José L McFaline-Figueroa, Lauren Saunders, Cole Trapnell, and Sreeram Kannan. Inferring causal gene regulatory networks from coupled single-cell expression dynamics using scribe. *Cell systems*, 10(3):265–274, 2020.

[28] Pierre-Cyril Aubin-Frankowski and Jean-Philippe Vert. Gene regulation inference from single-cell rna-seq data with linear differential equations and velocity inference. *Bioinformatics*, 36 (18):4774–4780, 2020.
